# Xpert Ultra testing of blood in severe HIV-associated tuberculosis to detect and measure *Mycobacterium tuberculosis* blood stream infection: a diagnostic and disease biomarker cohort study

**DOI:** 10.1016/S2666-5247(22)00062-3

**Published:** 2022-07

**Authors:** Linda Boloko, Charlotte Schutz, Nomfundo Sibiya, Avuyonke Balfour, Amy Ward, Muki Shey, Mark P Nicol, Rosie Burton, Robert J Wilkinson, Gary Maartens, Graeme Meintjes, David A Barr

**Affiliations:** a Wellcome Centre for Infectious Diseases Research in Africa, Institute of Infectious Disease and Molecular Medicine, University of Cape Town, Cape Town, South Africa; b Department of Medicine, University of Cape Town, Cape Town, South Africa; c The Francis Crick Institute, London, UK; d Division of Infection and Immunity School of Biomedical Sciences, University of Western Australia, Perth, Australia; e Division of Medical Microbiology, National Health Laboratory Services, Groote Schuur Hospital, Cape Town, South Africa; f Division of Clinical Pharmacology, Department of Medicine, University of Cape Town, Cape Town, South Africa; g Department of Infectious Disease, Imperial College, London, UK; h Institute of Infection and Global Health, University of Liverpool, Liverpool, UK; i Khayelitsha Hospital, Department of Medicine, Cape Town, South Africa

## Abstract

**Background:**

*Mycobacterium tuberculosis* bloodstream infection is a leading cause of death in people living with HIV and disseminated bacillary load might be a key driver of disease severity. We aimed to assess Xpert MTB/RIF Ultra (Xpert Ultra) testing of blood as a diagnostic for *M tuberculosis* bloodstream infection and investigate cycle threshold as a quantitative disease biomarker.

**Methods:**

In this cohort study, we obtained biobanked blood samples from a large and well characterised cohort of adult patients admitted to hospital in Western Cape, South Africa with suspected HIV-associated tuberculosis and a CD4 count less than 350 cells per μL. Patients already receiving antituberculosis therapy were excluded. Samples were obtained on recruitment within 72 h of admission to hospital, and patients were followed up for 12 weeks to determine survival. We tested the biobanked blood samples using the Xpert Ultra platform after lysis and wash processing of the blood. We assessed diagnostic yield (proportion of cases detected, with unavailable test results coded as negative) against a microbiological reference, both as a function of markers of critical-illness and compared with other rapid diagnostics (urine lipoarabinomannan and sputum Xpert). Quantitative blood Xpert Ultra results were evaluated as a disease biomarker by assessing association with disease phenotype defined by principal component analysis of 32 host-response markers. Prognostic value compared to other tuberculosis biomarkers was assessed using likelihood ratio testing of nested models predicting 12-week mortality.

**Findings:**

Between Jan 16, 2014, and Oct 19, 2016, of the 659 participants recruited to the parent study, 582 had an available biobanked blood sample. 447 (77%) of 582 met the microbiological reference standard for tuberculosis diagnosis. Median CD4 count was 62 (IQR 221–33) cells per μL, and 123 (21%) of participants died by 12-weeks follow-up. Blood Xpert Ultra was positive in 165 (37%) of 447 participants with confirmed tuberculosis by the microbiological reference standard, with a diagnostic yield of 0·37 (95% CI 0·32–0·42). Diagnostic yield increased with lower CD4 count or haemoglobin, and outperformed urine lipoarabinomannan testing in participants with elevated venous lactate. Quantitative blood Xpert Ultra results were more closely associated with mortality than other tuberculosis biomarkers including blood culture, and urine lipoarabinomannan, or urine Xpert (all p<0·05). A principal component of clinical phenotype capturing markers of inflammation, tissue damage, and organ dysfunction was strongly associated with both blood Xpert-Ultra positivity (associated with a SD increase of 1·1 in PC score, p<0·0001) and cycle threshold (*r*= −0·5; p<0·0001).

**Interpretation:**

Xpert Ultra testing of pre-processed blood could be used as a rapid diagnostic test in critically ill patients with suspected HIV-associated tuberculosis, while also giving additional prognostic information compared with other available markers. A dose–response relationship between quantitative blood Xpert Ultra results, host-response phenotype, and mortality risk adds to evidence that suggests *M tuberculosis* bloodstream infection bacillary load is causally related to outcomes.

**Funding:**

Wellcome Trust, National Institute of Health Fogarty International Center, South African MRC, UK National Institute of Health Research, National Research Foundation of South Africa.

**Translations:**

For the Xhosa and Afrikaans translations of the abstract see Supplementary Materials section.

## Introduction

Tuberculosis remains the leading reason for hospitalisation and death in people living with HIV.^1^ In severe HIV-associated tuberculosis, *Mycobacterium tuberculosis* blood stream infection (BSI) is both common and independently associated with mortality,^2^ and might therefore account for a substantial fraction of deaths in people living with HIV. However, *M tuberculosis* BSI has received less research attention than other forms of tuberculosis, at least in part because mycobacterial blood cultures are unavailable in most high-burden settings, and have limited diagnostic value because median time to positivity is longer than median time from admission to death in fatal cases of *M tuberculosis* BSI.^3^ As a result, while bloodstream dissemination is a cardinal feature of severe HIV-associated tuberculosis, it is seldom diagnosed in clinical practice or measured in research studies. The ability to rapidly identify *M tuberculosis* BSI might have diagnostic use in critically ill people living with HIV and could facilitate research into this neglected condition.

**Research in context**

**Evidence before this study**

To identify research studies reporting use of nucleic acid amplification technology (NAAT) on peripheral blood as a tuberculosis diagnostic, we searched PubMed and Scopus, without language restriction from database inception to Dec 12, 2020, using the terms “tuberculosis” AND (“blood” OR “mycobacter*emia” OR “blood stream infection” OR “bacter*emia” OR “bacill*emia”) AND (“NAAT” OR “PCR” OR “Xpert”) AND “diagnosis” (full systematic review and meta-analysis in appendix 3 [pp 17–33]). Since the 1990s dozens of uses of NAAT on blood to diagnose tuberculosis have been reported, with extreme variation in reported sensitivity (0–100%, 90% prediction interval 9–97%) not discernibly related to plausible biological covariates such as measures of disease spectrum or severity (eg, HIV status, patient setting, prevalence of prevalence of TB blood culture positive disease) or technical covariates (volume of blood and blood pre-processing methods). Most studies used in-house PCR protocols and are poorly reported with high risk of bias. Promising results in smaller studies have not been replicated in larger studies or studies with low risk of bias, or in studies using scalable, commercially available PCR platforms.

To identify studies reporting measurement of bacillary load in blood and relating it to patient outcomes or host-response variables we searched PubMed without language restriction from database inception to Sept 20, 2021, using the terms “tuberculosis” AND (“mycobacter*emia” OR “blood stream infection” OR “bacter*emia” OR “bacill*emia” OR “sepsis” OR “septic*emia”). We found one individual patient data meta-analysis, which systematically reviewed studies recruiting people living with HIV and performing mycobacterial blood culture and the meta-analysis reported that *Mycobacterium tuberculosis* blood stream infection was associated with an adjusted hazard ratio of 2·5 (95% CI 2·1–3·1) for 30-day death in patients with HIV-associated tuberculosis. We found one study that directly quantified *M tuberculosis* blood stream infection in nine patients using colony-forming unit counting, finding statistically non-significant negative correlation with CD4 count, and no association with mortality. Of note, blood or total body bacillary load was discussed as a potentially important but unmeasured variable in multiple studies, and is regarded as a key determinant of post-primary tuberculosis by tuberculosis pathologists working in the pre-antimicrobial era.

**Added value of this study**

Several small studies using in house PCR methods have suggested that rapid diagnosis of tuberculosis might be possible using NAAT on patient blood samples. Using a simple novel red blood cell lysis and wash method, we show that the WHO-endorsed Xpert MTB/RIF platform can be used to diagnose *M tuberculosis* blood stream infection in hospitalised patients with HIV-associated tuberculosis. Because the method is simple and Xpert MTB/RIF technology is widely available, our findings can be operationalised in numerous high burden settings. Our results also suggest which patients clinicians should consider for blood Xpert-Ultra testing: those who are too unwell to produce sputum or urine or those with sepsis or a raised venous lactate.

Further, this method allows measurement of blood bacillary load using cycle threshold values, which in turn gives additional prognostic information compared to other markers of bacillary dissemination. Markers of dissemination have been linked to prognosis in tuberculosis infection by previous studies, and bacterial load is widely hypothesised to be a key determinant of clinical phenotype, both specifically in tuberculosis infection, and in sepsis literature more generally. Because, in addition to detection, our method allows systematic quantification of tuberculosis dissemination, we were able to show a dose-response association between blood bacilli load and clinical phenotype. This gives direct evidence for a causal relationship between bacilli load and host response.

**Implications of all the available evidence**

Earlier reports that NAAT applied to blood can be used to diagnose tuberculosis have now been replicated in a large study using a protocol deliverable in routine clinical laboratories. Quantification of *M tuberculosis* blood stream infection using blood Xpert-Ultra might be valuable as a disease biomarker.

Many patients admitted to hospital with HIV-associated tuberculosis meet sepsis criteria,^4^ and tuberculosis is the most frequent microbiological diagnosis in patients with sepsis in high HIV-burden settings.^5^, ^6^ “A dysregulated host response to infection”^7^ is the consensus definition of sepsis,^7^ but the pathophysiological basis of this immune dysregulation remains incompletely defined. Hallmark host-responses characterising sepsis^8^, ^9^ are also found in life-threatening HIV-associated tuberculosis disease, including concurrent inflammatory and immunosuppressive signalling,^4^, ^10^ coagulation and endothelial activation,^11^, ^12^ innate cell activation and dysfunction,^4^, ^13^ and lymphopenia and exhaustion of T-cell responses.^4^, ^10^, ^14^ Microbiological data (eg, causative microbe and pathogen-burden) are notably absent from contemporary definitions of sepsis. By contrast, severity of tuberculosis infection has classically—from animal model^15^ and post-mortem studies^16^—been related to mycobacterial load, and specifically the “number of bacilli reaching the bloodstream and multiplying in the tissues”.^17^ The absence of tools to directly and systematically measure bacillary load antemortem has been a fundamental limitation of modern clinical studies investigating host response in severe HIV-associated tuberculosis.

Xpert MTB/RIF (Xpert; Cepheid, Sunnyvale, CA, USA) testing of sputum can detect most pulmonary tuberculosis in people living with HIV,^18^ and the next-generation Xpert MTB/RIF Ultra (Xpert-Ultra) test has further increased sensitivity.^19^ Sputum Xpert testing results in earlier institution of therapy, although evidence of impact on mortality is lacking.^20^ In critically ill inpatients, sputum-based diagnostics are limited by the frequent inability to obtain sputum.^5^

These limitations of sputum Xpert have led to calls to develop and validate rapid diagnostic tests targeted specifically at inpatients with sepsis and suspected *M tuberculosis* BSI,^3^ in whom delayed diagnosis is most dangerous.^2^ Early promising reports of *M tuberculosis* detection in blood using nucleic acid amplification tests (NAAT)^21^ have not been replicated in other studies,^22^ perhaps because the in-house assays developed had variable technical performance, with no standardised method emerging for validation in routine care settings. Subsequent attempts at using the commercially available Xpert-Ultra on blood as a rapid diagnostic for *M tuberculosis* BSI reported poor sensitivity,^23^, ^24^ likely due in part to PCR inhibition by blood components. Xpert testing of blood remains an attractive target. Xpert is a widely available platform capable of rapid diagnosis of both MTB and rifampicin resistance, blood is a major site of disease in severe HIV-associated tuberculosis, and is accessible in patients who are severely ill.

We used a simple pre-processing method to detect and quantify *M tuberculosis* in biobanked blood samples from a large cohort of patients hospitalised with presumed HIV-associated tuberculosis using Xpert-Ultra, with two objectives. First, we assessed diagnostic utility, both in comparison to other rapid diagnostics, and assessing how diagnostic use relates to markers of critical illness. Second, we assessed quantitative blood Xpert-Ultra results as a potential prognostic and disease biology biomarker, testing the hypothesis that *M tuberculosis* BSI bacillary load has a dose-res–ponse relationship with mortality and host–response phenotype.

## Methods

### Study design and participants

We evaluated Xpert Ultra on biobanked whole blood specimens from a well characterised cohort of participants hospitalised with suspected HIV-associated tuberculosis.^4^ The parent study^4^ recruited people living with HIV who's CD4 count was less than 350 cells per μL and were admitted to Khayelitsha Hospital, Cape Town, South Africa with suspected tuberculosis between Jan 16, 2014, and Oct 19, 2016, excluding patients already receiving antituberculosis therapy (appendix 3 p 4). All had baseline testing that was prospectively planned for tuberculosis within 72 h of admission, and were followed up for 12 weeks.^4^ For this analysis we selected patients according to the availability of biobanked blood specimens from the day of recruitment, and excluded patients without at least one test result for the diagnostic reference standard (defined below). This study was approved by the University of Cape Town Human Research Ethics Committee (HREC 057/2013). Written informed consent was obtained from all participants.

### Procedures

Sample collection occurred on the day of recruitment. If not already obtained by hospital staff, an experienced operator with access to sputum induction facilities attempted collection; all sputum was sent for liquid culture and Xpert. Urine Xpert Ultra was done on a centrifuged urine pellet as previously described.^25^ 5 mL of whole blood was cultured in Myco/F-Lytic (Becton-Dickinson Biosciences, Sparks, MD 21152 USA) bottles for 42 days or longer. All microbiology tests were done by the National Health Laboratory Services (appendix 3 p 4). Urine lipoarabinomannan (LAM) testing was done retrospectively on frozen samples using the Alere Determine TB-LAM test (Abbott Laboratories, Lake Bluff, USA). In a random subset of participants, soluble immune mediators were quantified in plasma derived from 4 mL whole blood samples stored at −80°C using Bio-Plex Pro-TM Human Cytokine Standard 27-Plex kit (Bio-Rad Laboratories, Watford, UK). Whole blood in 7 mL EDTA tubes (3–7 mL) were stored at −80°C.

Thawed 3–7 mL whole blood samples were made up to a volume of 45 mL with sterile red blood cell lysis-buffer (155 mM NH_4_Cl; 12 mM NaHCO_3_; 0·1 mM EDTA), left on the bench at room temperature for 30 min, centrifuged at 3500 g for 25 min, and the pellet resuspended in 45 mL water. This was re-incubated and centrifuged as above, with approximately 2·5 mL pellet residual volumes refrozen at −80°C for subsequent batch processing. Thawed samples were resuspended in 12·5 mL volume of sterile water, centrifuged at 3500 g for 25 min, and residual pellet volume of 0·7 mL mixed with 1·5 mL Xpert Ultra sample reagent (Cepheid, Sunnyvale, CA, USA). This was incubated at room temperature for 15 min, before transfer to Xpert Ultra cartridges (Cepheid, Sunnyvale, CA, USA). A summary of the method development is provided in appendix 3 (pp 6–7). Tests were done by biomedical scientists who were masked to clinical data.

We defined sensitivity as the number of participants with a positive result on the index test divided by total number of participants with: (1) a valid index test result; and (2) tuberculosis diagnosis confirmed by a strict microbiological reference standard of *M tuberculosis* culture from any site or positive Xpert from any site other than blood.

We defined diagnostic yield as number of participants with a positive result on the index test divided by the total number of participants who met an extended microbiological reference standard: any positive MTB culture or Xpert from any site, or positive urine LAM. Participants with a missing test result due to an inability to obtain samples or technical failure of the index test were included as negative results in the numerator.

Positive and negative blood Xpert-Ultra results in patients who were negative by all other tests in the extended microbiological reference standard were defined as false positive and true negative, respectively; patients with less than two valid test results in the extended microbiological reference standard were excluded from this specificity analysis.

### Statistical analysis

Participant characteristics were summarised using frequency and proportions, median and IQR, Fisher's test and Kruskal Wallis test. To explore the patient groups in which blood Xpert Ultra testing might be most useful, sensitivity and diagnostic yield were calculated in the overall population and different strata defined a priori by CD4 count less than 100 cells per μL, haemoglobin less than 8 g/dL, and venous lactate more than 2·5 mmol/L. Diagnostic yield was modelled as a continuous function of CD4 count, haemoglobin concentration, and lactate concentration using a Locally Estimated Scatterplot Smoothing regression, with confidence intervals derived from 1000 bootstraps. Intersections between tuberculosis detection on rapid diagnostic tests were explored using set-intersection, euler diagrams, and mosaic plots with covariance compared, including culture results, using Cohen's kappa and factor analysis. Quantitative results including cycle threshold values and time to positivity of cultures were compared using correlation plots and rank-correlation test. Association of missing urine sample with venous lactate was assessed by logistic regression in a post-hoc analysis to explore the finding of low urine LAM diagnostic yield in patients with hyperlactataemia.

Minimum rpoB probe cycle threshold value was extracted using a custom R script from raw text files exported from the Xpert software as a value. We used IS1081-IS6110 cycle threshold values to impute blood Xpert-Ultra cycle threshold values for so-called trace positive samples, using a restricted cubic spline model with three knots to model the relationship between rpoB probe and IS1081–IS6110 cycle threshold values (appendix 3 p 8).

Blood Xpert Ultra results were assessed for association with 12-week mortality both as a qualitative result (positive or negative, in patients with confirmed HIV-associated tuberculosis) by Fisher's exact test, and by cycle threshold value (in the stratum of participants with a positive result) by logistic and Locally Estimated Scatterplot Smoothing regression. Equivalent analyses of tuberculosis blood culture results, sputum Xpert, and urine Xpert results were made for comparison. Blood Xpert-Ultra cycle threshold values were categorised by tertile to give an ordinal scale ranging from 0 (negative test) to 3 (positive test with lowest cycle threshold values), and assessed for association with 12-week mortality by Kaplan Meier curves and Cox proportional hazards regressions estimating hazard ratios with 95% CIs. Equivalent models were made using quick sequential organ failure assessment scores, urine LAM results, and ordinal versions of tuberculosis blood culture, sputum Xpert, and urine Xpert results. To formally compare the strength of association with mortality for these variables, nested models were made and compared using likelihood ratio tests, assessing if these variables added value to models that included blood Xpert Ultra, and if blood Xpert Ultra added value to models that included these variables, at a level of significance of less than 0·05.^26^ To allow these nested comparisons, missing observations of sputum-based and urine-based diagnostics were multiple imputed using multivariate imputation by chained equations package in R (version 4.0.2), with likelihood ratio statistics averaged across ten imputed datasets.

Association of blood Xpert Ultra with 32 clinico-immunological variables (selected a priori based on known mortality association in this cohort)^4^ were assessed; blood Xpert Ultra results were assessed on an ordinal scale as above, and association assessed by rank correlation tests. Q-values representing correction of p values for multiple comparisons using Benjamini-Hochberg procedure to limit false discovery rate were derived. Principal components analysis with varimax rotation was performed and the resulting two-dimensional representation of host-response phenotype related to blood Xpert Ultra cycle threshold values using both Locally Estimated Scatterplot Smoothing and linear regression, with derived Pearson's r correlation coefficient.

All analysis was done using RStudio v1.2.5033, with code and blood Xpert Ultra data available at GitHub. Further meta-data available on request from the corresponding author. STARD checklist is in the supplementary appendix 3 (pp 1–2).

### Role of the funding source

The funder of the study had no role in study design, data collection, data analysis, data interpretation, or writing of the report.

## Results

582 (83%) of 659 participants recruited to the parent study had biobanked whole blood available and were included in the current study (table 1). All 659 participants in the parent study were successfully venesected; the 77 missing samples were used in other studies, and were considered missing completely at random (appendix 3 p 8; appendix 3 pp 15–16) and not included in the diagnostic utility denominator. Tuberculosis was confirmed in 424 (73%) participants by the strict microbiological reference standard, and 447 (77%) by extended reference. Participants had a median of six (IQR 5–8) baseline tuberculosis diagnostic tests with valid results and therefore no participants were excluded due to incomplete reference standard. Median CD4 count was 62 cells per μL (IQR 221–33) and 123 (21%) participants died by 12 weeks follow-up (table 1).

**Table 1:** Patient characteristics

			**Blood Xpert Ultra negative (n=413)**†	**Blood Xpert Ultra positive (n=165)**†	**Total (N=582)**†	**p value**†
Age, years			36·3 (31·0–44·0)	36·0 (31·0–43·7)	36·3 (40·0–44·0)	0·8500
CD4 count, cells per μL			86 (34–160)	25 (8–60)	62 (22–133)	<0·0001
Heart rate, beats per min			102·5 (92–117)	111·0 (98–123)	104·0 (94–120)	<0·0001
Venous Lactate, mmol/L			1·7 (1·2–2·3)	2·1 (1·5–3·1)	1·8 (1·3–2·5)	<0·0001
Haemoglobin, g/dL			9·3 (7·6–10·8)	8 (6·7–9·3)	8·8 (7·3–10·5)	<0·0001
Creatinine, mmol/L			76·0 (58–105)	95·0 (66–161)	78·5 (59–118)	<0·0001
C-reactive protein, mg/L			137·0 (75–225)	196·0 (130–251)	153·5 (87–232)	<0·0001
Sodium, mmol/L			130 (126–132)	127 (124–130)	129 (125–132)	<0·0001
Sex			..	..	..	0·0120
	Female		227 (55%)	76 (46%)	303 (52%)	..
	Male		186 (45%)	89 (54%)	279 (48%)	..
Antiretroviral status			..	..	..	<0·0001
	Missing observation		6	1	7	..
		Defaulted	81 (20%)	49 (30%)	133 (23%)	..
		Naive	150 (37%)	70 (43%)	220 (38%)	..
		On antiretroviral therapy	176 (43%)	45 (27%)	222 (39%)	..
Cough			..	..	..	0·8100
	Missing observation		13	8	21	..
	No		122 (31%)	52 (33%)	175 (31%)	..
	Yes		278 (70%)	105 (67%)	386 (69%)	..
Loss of appetite			..	..	..	0·0690
	Missing observation		14	12	26	..
	No		140 (35%)	44 (29%)	187 (34%)	..
	Yes		259 (65%)	109 (71%)	369 (66%)	..
Night sweats			..	..	..	0·2400
	Missing observation		18	12	30	..
	No		178 (45%)	67 (44%)	245 (44%)	..
	Yes		217 (55%)	86 (56%)	307 (56%)	..
Weight loss			..	..	..	0·8500
	Missing observation		16	11	27	..
	No		43 (11%)	15 (10%)	58 (10%)	..
	Yes		354 (89%)	139 (90%)	497 (90%)	..
Outcome 12 weeks			..	..	..	0·0008
	Died		70 (17%)	51 (31%)	123 (21%)	..
	Lost to follow-up		10 (2%)	2 (1%)	12 (2%)	..
	Survived		333 (81%)	112 (68%)	447 (77%)	..
Final diagnosis at end of study			..	..	..	<0·0001
	Tuberculosis		278 (67%)	163 (99%)	447 (77%)	..
	No alternative diagnosis made		74 (18%)	2 (1%)	76 (13%)	..
	Community acquired pneumonia		31 (8%)	0	31 (5%)	..
	Other opportunistic infection or malignancy		12 (3%)	0	12 (2%)	..
	Other bacteraemia		6 (2%)	0	6 (1%)	..
	Cryptococcus neoformans		5 (1%)	0	5 (1%)	..
	Pneumocystis jirovecii		5 (1%)	0	5 (1%)	..

Data are n (%) or median (IQR), unless otherwise stated. Xpert Ultra=Xpert MTB/RIF Ultra.

*Includes four patients with non-valid (failed) blood Xpert Ultra test.

† p values from Fisher's exact test for categorical variables and Kruskal Wallis test for numerical variables.

578 (99%) participants included had a valid blood Xpert Ultra result (n=4 failed tests), 519 (89%) had a valid urine LAM (n=0 failed tests, n=63 no urine sample obtained), and 445 (76%) had a valid sputum Xpert (n=2 failed tests, n=135 no sputum sample obtained).

**Figure 1:**
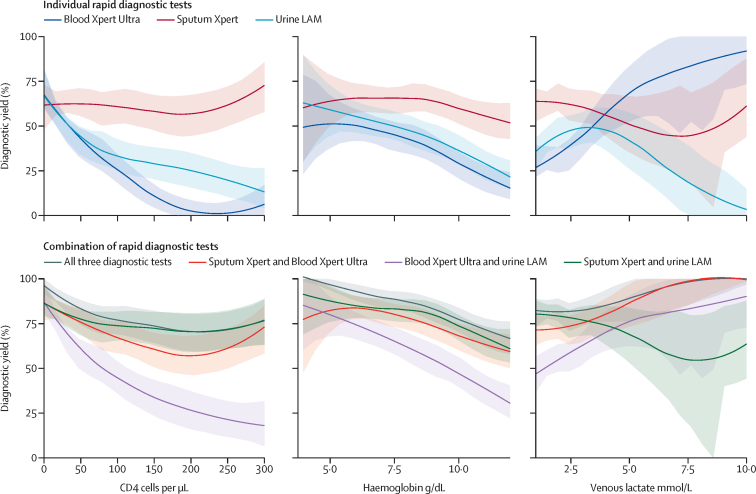
Diagnostic yield of individual and combination rapid diagnostic tests as a function of clinical variables Individual test (top row panels) and combinations of these tests (bottom row panels) diagnostic yield modelled as a function of three a priori specified patient variables: CD4 count (left column), haemoglobin (middle column), and venous lactate (right column). Plotted lines show a Locally Estimated Scatterplot Smoothing function fit to the proportion of tuberculosis cases identified by the diagnostic tests by different strata of the patient variables. Shaded areas indicate 95% CIs derived from 1000 bootstrapped resampling and refitting of the Locally Estimated Scatterplot Smoothing models. Xpert=Xpert MTB/RIF. Xpert Ultra=Xpert MTB/RIF Ultra. LAM=lipoarabinomannan.

165 (37%) of 447 participants with confirmed tuberculosis by the extended microbiological reference standard were positive on blood Xpert Ultra testing, giving a diagnostic yield of 0·37 (95% CI 0·32–0·42), which was lower than the diagnostic yield of sputum Xpert (0·62; 0·57–0·66) and urine LAM (0·43; 0·38–0·47). By comparison, blood Xpert Ultra did relatively well in the prespecified subgroups (ie, patients with CD4 count less than 100 cells per μL, haemoglobin less than 8 g/μL, venous lactate more than 2·5 mmol/L, and in those who died; table 2; figure 1). Diagnostic yield of blood Xpert-Ultra approximated that of urine LAM across CD4 count range 0–100 cells per μL, and for patients with haemoglobin 5–9 g/dL. However, a significant divergence between the two was seen with rising venous lactate concentrations: blood Xpert-Ultra did increasingly well, whereas diagnostic yield of urine LAM decreased (figure 1). Hyperlactataemia was associated with inability to obtain a urine sample (odds ratio [OR] 1·8 for missing urine sample for 1 log increase in venous lactate; 1·4–4·4, p=0·003). Combining sputum-Xpert with either urine LAM or blood Xpert-Ultra was broadly equivalent (figure 1).

**Table 2:** Sensitivity and diagnostic yield in whole cohort and subgroups

		**Valid test**	**Confirmed tuberculosis by strict reference standard**	**True positive**	**Sensitivity**	**95% CI**
**Diagnostic test**						
Whole cohort (n=582)						
	Alere LAM	519 (89·2%)	375	171	0·46	0·40–0·51
	Blood Xpert Ultra	578 (99·3%)	423	161	0·38	0·33–0·43
CD4 count less than 100 cells per μL (n=375)						
	Alere LAM	329 (87·7%)	254	145	0·57	0·51–0·63
	Blood Xpert Ultra	372 (99·2%)	288	145	0·50	0·44–0·56
Haemoglobin less than 8 g/dL (n=207)						
	Alere LAM	175 (84·5%)	144	89	0·62	0·53–0·70
	Blood Xpert Ultra	206 (99·5%)	170	80	0·47	0·39–0·55
Lactate less than 2·5 g/dL (n=142)						
	Alere LAM	119 (83·8%)	97	49	0·51	0·40–0·61
	Blood Xpert Ultra	139 (97·9%)	115	59	0·51	0·42–0·61
Died by week 12 (n=123)						
	Alere LAM	101 (82·1%)	74	39	0·53	0·41–0·64
	Blood Xpert Ultra	121 (98·4%)	89	49	0·55	0·44–0·65
qSOFA score 2 or more (n=124)						
	Alere LAM	108 (87·1%)	77	39	0·51	0·39–0·62
	Blood Xpert Ultra	124 (100%)	87	44	0·51	0·40–0·61
**Diagnostic yield**						
Whole cohort						
	Sputum Xpert	582	447	275	0·62	0·57–0·66
	Alere LAM	582	447	162	0·43	0·38–0·47
	Tuberculosis blood culture	582	447	190	0·36	0·32–0·41
	Blood Xpert Ultra	582	447	165	0·37	0·32–0·42
CD4 less than 100 cells per μL						
	Sputum Xpert	374	300	185	0·62	0·56–0·67
	Alere LAM	374	300	152	0·51	0·45–0·56
	Tuberculosis blood culture	375	301	146	0·49	0·43–0·54
	Blood Xpert Ultra	374	300	148	0·49	0·44–0·55
Haemoglobin less than 8 g/dL						
	Sputum Xpert	205	177	112	0·63	0·56–0·70
	Alere LAM	205	177	92	0·52	0·44–0·59
	Tuberculosis blood culture	207	179	78	0·44	0·36–0·51
	Blood Xpert Ultra	205	177	82	0·46	0·39–0·54
Lactate more than 2·5 g/dL						
	Sputum Xpert	140	122	67	0·55	0·46–0·64
	Alere LAM	140	122	52	0·43	0·34–0·52
	Tuberculosis blood culture	142	124	63	0·51	0·42–0·60
	Blood Xpert Ultra	140	122	60	0·49	0·4–0·58
Died by week 12						
	Sputum Xpert	123	96	53	0·55	0·45–0·65
	Alere LAM	123	96	48	0·45	0·35–0·55
	Tuberculosis blood culture	123	96	43	0·50	0·40–0·60
	Blood Xpert Ultra	123	9	51	0·53	0·43–0·63
qSOFA score 2 or more						
	Sputum Xpert	124	92	51	0·55	0·45–0·66
	Alere LAM	124	92	44	0·48	0·37–0·58
	Blood Xpert Ultra	124	92	44	0·48	0·37–0·58

LAM=lipoarabinomannan. Xpert=Xpert MTB/RIF. Xpert Ultra=Xpert MTB/RIF Ultra. qSOFA=quick sepsis-related organ failure assessment.

Two blood Xpert-Ultra positive participants were negative on all other available tuberculosis diagnostic tests (specificity 0·98; 95% CI 0·94–1·0) but did present clinical and radiological evidence of tuberculosis (appendix 3 p 16). All ten patients with bloodstream infection from an alternative pathogen in the non-tuberculosis group (five *Streptococcus pneumoniae* and five Gram-negative BSI) were negative by blood Xpert Ultra testing.

**Figure 2:**
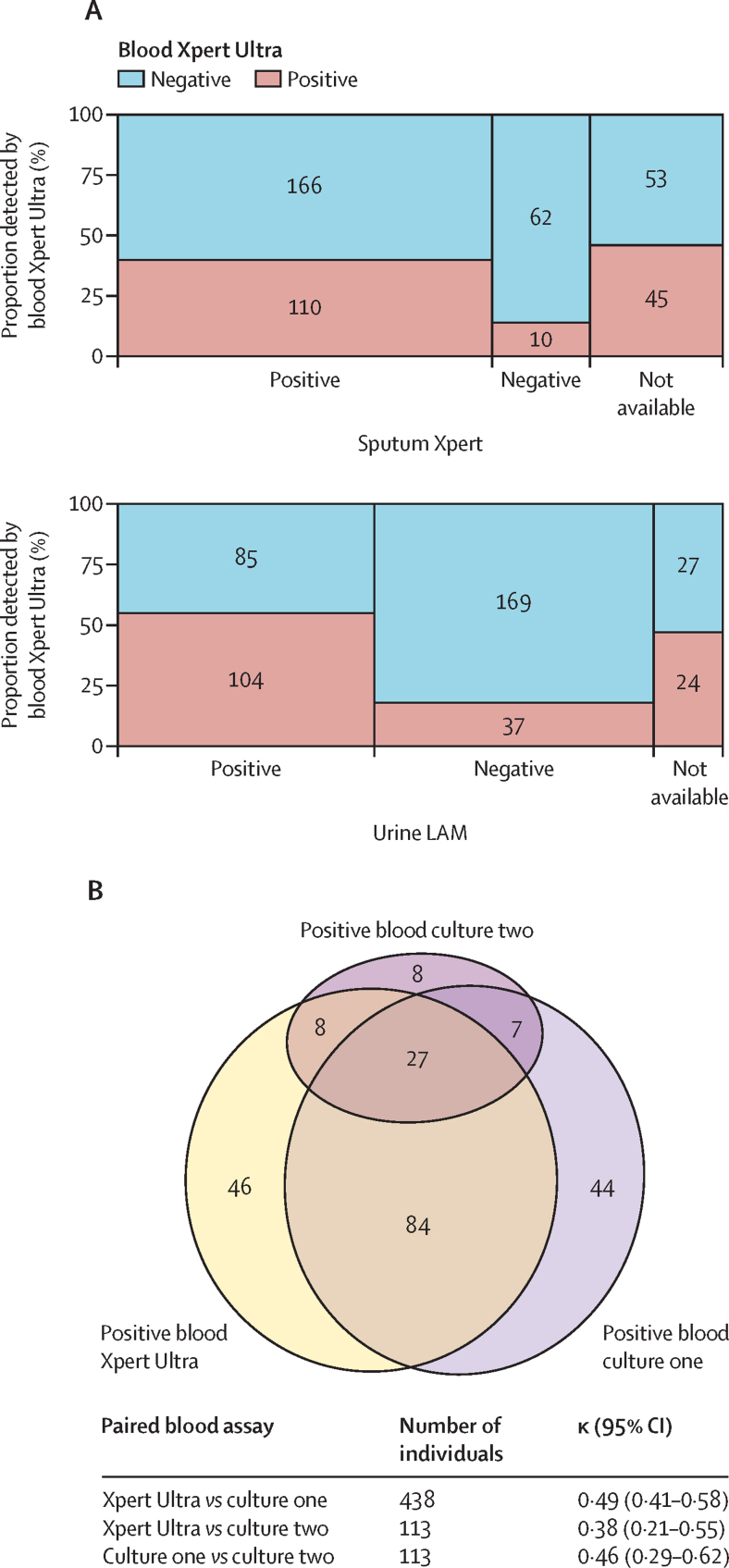
Qualitative and quantitative associations between tuberculosis detection modalities (A) Detection of *Mycobacterium tuberculosis* by blood Xpert Ultra in participants with missing sputum and urine samples. (B) Intersections between *M tuberculosis* bloodstream infection diagnostics. Blood Xpert Ultra was positive in 111 (69%) of 162 patients who had tuberculosis recovered from their first blood culture. Out of 113 patients who also had a second tuberculosis blood culture 50 (44%) were positive; blood Xpert Ultra was positive in 35 of 50 of these. Agreement (measured by Cohen's kappa) between blood Xpert Ultra and blood culture one was the same as agreement between pairs of blood cultures in the same patient. Xpert Ultra=Xpert MTB/RIF Ultra. LAM=lipoarabinomannan.

Blood Xpert Ultra was positive in 45 (46%) of 98 participants with missing sputum and 24 (47%) of 51 participants with missing urine (figure 2A). Blood Xpert Ultra was positive in 111 (69%) of 162 participants with a positive tuberculosis blood culture, with moderate agreement between the two modalities (Cohen's kappa 0·49; 95% CI 0·41–0·58) equivalent to agreement between two blood cultures (figure 2B). In general, covariance of positive rapid diagnostic tests was related to compartment sampled, with blood and urine samples having closer agreement to each other relative to sputum diagnostics (irrespective of detection method; appendix 3 pp 9).

**Figure 3:**
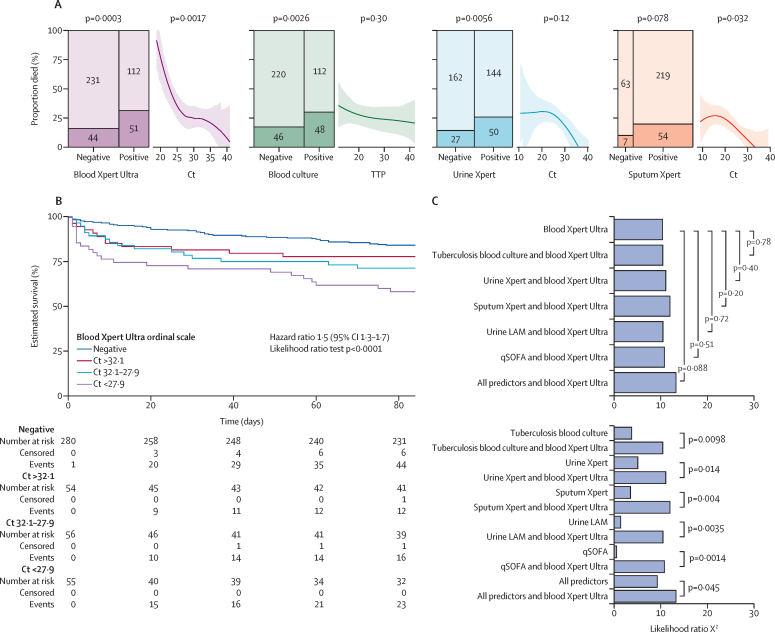
The association of blood Xpert Ultra with 12-week mortality and comparison to other diagnostic tests as predictors of mortality (A) Mosaic plots of two by two cross-tabulation of test results (positive or negative) and mortality for the four diagnostics, blood Xpert Ultra, tuberculosis blood culture, urine Xpert, and sputum Xpert. Y-axis indicates proportion who died by day 84 follow-up, area of each cell proportional to numbers in cross-tabulation category. p values from Fisher's exact test. Also shown are estimated proportions of patients who died by Ct (Xpert tests) or time to positivity (blood culture) for those patients with positive tests. A Locally Estimated Scatterplot Smoothing function fit is shown by line with 95% CI for the fit derived from 1000 bootstraps; OR_IQR_ indicates the OR for a decrease in Ct or time-to-positivity of one IQR (the IQR effect size) with associated p value derived by logistic regression. (B) Kaplan-Meier plot showing survival by blood Xpert Ultra result on an ordinal scale, in which positive results are further categorised by Ct value cut-offs based on observed tertiles, giving four ordinal categories. HR for a one unit increase in ordinal category, with 95% CI from a Cox proportional hazards model fit to this data. Goodness-of-fit test for violation of proportional hazards assumption, p>0·05. (C) Predictive value of blood Xpert Ultra result—on ordinal scale as per (B). Formally compared with other variables from the set (tuberculosis blood culture, urine Xpert, sputum Xpert, urine LAM, and qSOFA score) using likelihood ratio tests. The addition of each of these variables to blood Xpert Ultra results, either individually or in combination, did not significantly improve model fit over blood Xpert Ultra alone, as indicated by p values from likelihood ratio tests with 1 degree of freedom in the top panel. By contrast, addition of blood Xpert Ultra result to each of these variables as an individual predictor or when all were combined did improve model fit in all cases, as indicated by p values from likelihood ratio tests in the lower panel. Ct=cycle threshold. HR=hazard ratio.LAM=lipoarabinomannan. . LRT p value=likelihood ratio test of model fit. OR=odds ratio. HR=hazard ratio. Xpert=Xpert MTB/RIF. Xpert Ultra=Xpert MTB/RIF Ultra. qSOFA=quick sepsis-related organ failure assessment.

A positive blood Xpert-Ultra result was associated with an OR for 12-week mortality of 2·39 (95% CI 1·5–3·9) compared with 2·08 for tuberculosis blood culture (1·3–3·4) and 2·08 for urine Xpert (1·2–3·6; figure 3A). Blood Xpert-Ultra cycle threshold value also correlated with mortality, showing a larger effect size than other quantitative tuberculosis markers (figure 3A): predicted probability of death was 0·25 (0·16–0·36) with blood Xpert-Ultra cycle threshold of 28, rising to 0·57 (0·42–0·70) with cycle threshold of 22. To make use of all the information generated by the tests, test positivity and quantitative read-outs (cycle threshold or time-to-positivity) were combined on an ordinal scale, and used as a predictor variable (figure 3B). This showed that blood Xpert Ultra was more strongly associated with mortality than any of: tuberculosis blood culture, urine Xpert, sputum Xpert, urine LAM, quick sequential organ failure assessment score, or these five variables combined as a multivariable predictor (figure 3C).

In participants with confirmed tuberculosis, 31 (99%) of 32 of the clinical-immunological variables correlated with blood Xpert-Ultra after correction for multiple testing (appendix 3 pp 10–12). Markers of acute inflammation, innate cell chemotaxis, coagulation system activation, lymphocyte counts, and interleukin-1 receptor antagonist were most strongly associated.

**Figure 4:**
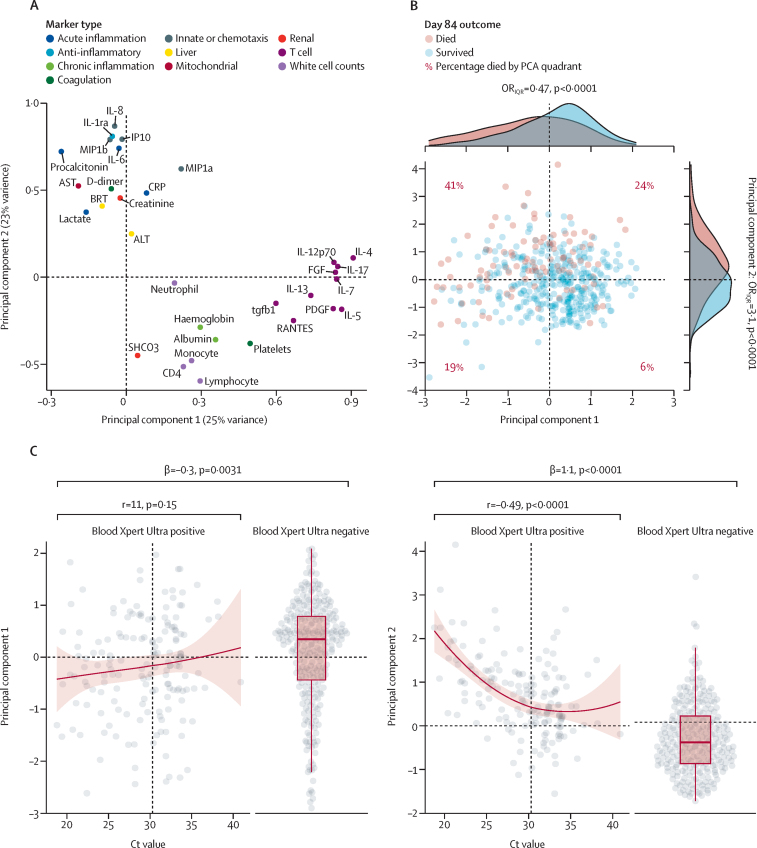
Major axes of covariance in clinic-immunological variables and their relationship to blood Xpert Ultra results Principal components analysis of 32 clinic-immunological variables using varimax rotation done on 447 patients with confirmed tuberculosis. (A) Loadings of the 32 variables on first two principal components, which together capture 48% of total variance. (B) Individual patients' principal components 1 and principal components 2 scores, by day 84 outcome. Density histograms show distributions of patients' principal components 1 and principal components 2 scores by day 84 outcome. OR_IQR_ indicates the OR for mortality associated with a one IQR increase in principal components score (the IQR effect size) with associated p value derived by logistic regression. Scatter-plot shows mortality outcome mapped onto the two-dimensional space defined by principal components 1 and principal components 2 scores. (C) Distribution of principal components 1 and 2 scores by blood Xpert Ultra Ct value, and in patients with a negative blood Xpert Ultra result. A locally estimated scatterplot smoothing fit regressing principal components score on Ct value is shown in the strata of patients with a positive blood Xpert Ultra, with 95% CI indicated by red shaded area, as well as a correlation coefficient and associated p value for a linear regression. Distribution of principal components score in blood Xpert Ultra negative patients is shown, with β coefficient from regressing principal components score on blood Xpert Ultra result (indicating the average difference in principal components score between blood Xpert Ultra positive and negative patients). Ct=cycle threshold.

A substantial proportion host-response variation was captured in two principal components: variance in T-cell associated mediators on principal components 1, markers of acute inflammation on principal components 2, and blood cell counts loading partially on both axes (figure 4A). 12-week mortality risk mapped closely to this 2-dimensional representation of clinical phenotype: the quarter of participants with below average principal components 1 score and above average principal components 2 score had an OR for mortality of 11 compared with the quarter of participants with above average principal components 1 and below average principal components 2 scores (upper-left principal component analysis quadrant versus lower-right principal component analysis quadrant, figure 4B: OR 11 (95% CI 4·7–29; p<0·0001). In turn, blood Xpert Ultra positivity was associated with a 0·3 SD decrease in principal components 1 score (p=0·003), and a 1·1 SD increase in principal components 2 score (p<0·0001; figure 4C). Within the stratum of participants with a positive blood Xpert Ultra, cycle threshold value was not significantly related to principal components 1, but showed strong correlation with principal components 2 score (r=–0·49; p<0·0001; figure 4C): 68 (91%) of 75 participants with blood Xpert-Ultra cycle threshold value below the median had an above-average principal components 2 score.

## Discussion

Detection of *M tuberculosis* blood stream infection using the described protocol gives more prognostic information in severe HIV-associated tuberculosis than previously available markers of tuberculosis dissemination. Accordingly, blood Xpert Ultra has greatest diagnostic utility in patients who are critically ill: compared with Alere urine LAM, blood Xpert Ultra had similar diagnostic yield in participants with low CD4 counts, and higher yield in participants with raised venous lactate. Combination of rapid diagnostics has better yield than individual tests, and we suggest blood Xpert Ultra has value in patients too unwell to produce sputum. Blood Xpert-Ultra cycle threshold value has a robust dose-response relationship with disease phenotype and mortality risk: giving evidence that blood bacillary load has an important, and potentially causal, role in HIV-associated tuberculosis outcomes.

We optimised the preprocessing of blood requiring only low-cost reagents, combined with the Xpert Ultra platform, and report higher sensitivity (38%) than previous studies.^23^, ^24^ The method is accessible in low-resource settings where the Xpert platform is available. This is in contrast to previous studies using in-house PCR protocols that are not scalable to routine clinical labs.^21^, ^22^ Ease of blood sampling in clinical settings, same-day results, rifampicin-resistance reporting, and prognostic value, are additional benefits.

*M tuberculosis* BSI is common, under-recognised, and related to high mortality-risk when initiation of anti-tuberculosis therapy is delayed, compared with other forms of HIV-associated tuberculosis.^2^, ^4^ Tuberculosis blood culture is unavailable in most settings, and prolonged time-to-detection decreases its use in patients who are critically ill. Blood Xpert-Ultra could replace tuberculosis blood culture as the tests have equivalent sensitivities. The ability to rapidly identify patients with *M tuberculosis* BSI also strengthens the rationale for developing a dedicated evidence base for treatment of this critical condition.

Markers of dissemination in HIV-associated tuberculosis have been linked to adverse clinical status, sepsis, and mortality.^4^, ^27^ In this analysis of participants with advanced HIV-associated tuberculosis, blood Xpert-Ultra was more strongly associated with mortality than other bacillary load and dissemination markers (eg, tuberculosis blood culture, urine LAM, urine Xpert, and sputum Xpert). Risk of mortality strongly correlated with a low-dimensional representation of host–response phenotype, which in turn closely corresponded with blood Xpert Ultra results. Even within the stratum of participants with a positive blood Xpert Ultra test, we found clinical phenotype and mortality risk correlated with cycle threshold values as a quantitative read-out of *M tuberculosis* BSI. This robust dose–response relationship strengthens the evidence that blood bacillary load is a major determinant of outcome in HIV-associated tuberculosis.

This insight resonates with classical understandings of tuberculosis pathophysiology, which held bacillary load to be the major determinant of disease severity.^15^, ^17^ Early 20th century tuberculosis pathologists understood blood stream dissemination to be characteristic in the natural history of symptomatic and asymptomatic post primary tuberculosis; more recently investigators have shown that tuberculosis can be detected in CD34-positive cells recovered from peripheral blood during latent infection.^28^ We suggest that blood bacillary load is a fundamental variable for interrogating dysregulated host-response in HIV-associated tuberculosis. Recent studies^12^, ^29^ of host response in severe HIV-associated tuberculosis and sepsis acknowledge that microbial load might be an important latent variable but have lacked the tools to measure it. We found the blood bacillary load dose–response relationship was most strongly seen with a host-response component capturing innate-cell, pro-inflammatory and anti-inflammatory, and tissue damage signals, rather than the other major axis of variation in clinical phenotype which appeared to represent T-cell dysfunction. We speculate that antimicrobial and host-directed interventions targeting each of these pathophysiological components could be synergistic. For example, anti-inflammatory strategies should be combined with optimised antimicrobial killing.

Although blood Xpert-Ultra cycle threshold value correlated with tuberculosis blood culture time-to-positivity, the former was more robustly associated with risk of mortality. Possible explanations for this include that time-to-positivity has more stochastic variation than Ct, that time-to-positivity reflects lag-time for the fastest-growing bacilli in the sample rather than directly reporting overall bacilli numbers,^30^ or that Xpert Ultra captures non-culturable bacilli. By extension, blood Xpert Ultra cycle threshold might be a more useful microbial load biomarker than time-to-positivity in future studies of severe HIV-associated tuberculosis.

Limitations of our analysis include retrospective use of biobanked samples: sample volumes varied and might not have been optimal for the blood Xpert Ultra protocol, which could have introduced noise and reduced sensitivity. Because all patients were recruited based on the high index of clinical suspicion for tuberculosis, relatively few samples were available from patients ultimately classified as not having tuberculosis, limiting precision of specificity estimates. Storage at −80°C might also have reduced sensitivity (appendix 3 p 13). The reported sputum diagnostic yield might be higher than clinical practice because of availability of sputum induction.

In conclusion, we report the use of Xpert Ultra on whole blood and show its use as a novel rapid diagnostic and prognostic biomarker for critically ill patients with HIV-associated tuberculosis. Blood Xpert Ultra is a useful tool for characterising MTB BSI, demonstrating dose–response association between blood bacillary load, and adverse clinical phenotype.

For **GitHub** see https://github.com/davidadambarr/blood_xpert_repo

## Declaration of interests

We declare no conflict of interest.

## Supplementary Materials

Xhosa translation of the abstract

Afrikaans translation of the abstract
