# Supplementary material for: Xpert Ultra testing of blood in severe HIV-associated tuberculosis to detect and measure Mycobacterium tuberculosis blood stream infection: a diagnostic and disease biomarker cohort study
Source: Lancet Microbe. 2022 Jul;3(7):e521–32. doi: 10.1016/S2666-5247(22)00062-3 (PMC9242865; doi:10.1016/S2666-5247(22)00062-3)
Supplement: Xhosa translation of the abstract [file mmc1.pdf]

THE LANCET
Microbe

Supplementary appendix 1

This translation in Xhosa was submitted by the authors and we reproduce it as supplied. It has not been peer reviewed. *The Lancet*'s editorial processes have only been applied to the original in English, which should serve as reference for this manuscript.

Le nguqulelo yesiXhosa iye yafakwa ngababhali yaye siyayikhupha kutsha njengoko inikelwe. Ayikahlolisiswa zezinye iingcali. Iinkqubo zohlelo zeThe Lancet ziye zenziwa kuphela kumbhalo wokuqala ongesiNgesi, yaye kuya kufuneka ukuba zisetyenziswe njengembekiselo yalo mbhalo-ngqangi.

iXpert Ultra ku vavanyo egazini lwe sifo sephepha esinxulumane nentsholongwane ka gawulayo ukufumanisa nokulinganisa usulelo lwe ntsholongwane i*Mycobacterium tuberculosis* egazini: uphando lo xilonga kunye nophawu lwesifo.

Imvelaphi: Intsholongwane *iMycobacterium tuberculosis* eyenza isifo sephepha, xa ithe yafumaneka egazini ihamba phambili ukubulala abantu abaphila nentsholongwane ka gawulayo. Isifo sephepha siqatha kubantu abafumanekisa benetsholongwane *iMycobacterium tuberculosis* esasazeke umzimba. Injongo yolu phando ku vavanya iXpert MTB/RIF Ultra (okanye Xpert Ultra) ukufumanisa intsholongwane yesifo sephepha egazini kwaye nokuncedisa kwayo ukubona ubunintsi bale ntsholongwane.

Iindlela zophando: Kolu phando, kusetyenziswe igazi le qela laba gulayo, ebeligcinwe ngononophelo emkhenkceni. Iqela eli likhethwe kubantu abadala, abagulayo, ababelaliswe esibhedlele esise Ntshona Koloni, eMzantsi Afrika, bekrokrelwa ngesifo sephepha esinxulumene ne ntsholongwane ka gawulayo, bena majonini omzimba (CD4 count) engaphantsi kwe 350 cells per µL. Abo bebesele beqalise unyango lwesifo sephepha bakhuphelwe ngaphandle ko phando. Igazi litsalwe ngaphambi kophela kwentsuku ezintathu zokulaliswa esibhedlele, izigulane zabonwa ukuyofika kwi nyanga ezintathu ukulandelisa inkqubo yempilo. Sivavanye igazi eligciniwe kumkhenkce sisebenzisa iXpert Ultra emveni kwenkqubo zohluza igazi. Sivavanye idiagnostic yield (inani labaguli elifumaniseke linentsholongwane yesifo sephepha nge Xpert Ultra) isalathiswa naba ithe yabonwa intsholongwane ngevavanyo ziqhelekileyo. Kwaye sisebenzise nezinye ezaziwayo indlela zovavanya intshologwane yesifo sephepha ukuze sithelekise iziphumo zeXpert Ultra nelipoarabinomannan yomchamo kunye ne Xpert esikhohleleni. Iziphumo ze Xpert Ultra zibonisa ukuba zinga setyenziswa ukulandela inkqubela yesifo. Kusetyenziswe ubuchwepheshe be principal component analysis ukubonisa ukuba impawu eziyi 32 ziyakwazi ukubonakalisa umntu one ntsholongwane yesifo sephepha. Ubume bokugula kwaba fumanikese intsholongwane yesifo sephepha egazini buthelekiswa nezinye ivavanyo nge likelihood ratio testing ekwazi ukuqikelala ukufa okanye ukuphila ngesifo sephepha enyangeni ezintathu ezizayo.

Iziphumo: Kusuka kwi 16 kweyoMqungu 2014 kuquka 19 kweyeDwarha 2016, kuba thathi nxaxheba abayi 659, kwafumaneka amagazi kwi 582. 447 (77%) ye 582 yafumaneka inesifo sephepha ngovavanyo ze salathiso. Umdi we CD4 count yaba ngu 62 (IQR 33-221) cells per µL. Abathathi nxanxeba abayi 123 (21%) basweleka emveni kwenyanga ezintathu zophando. iXpert Ultra egazini ifumanise intsholongwane kwi 165 (37%) ye 447 efunyaniswe inesifo sephepha ngovavanyo ze salathiso, ufumaniso idiagnostic yield ka 0·37 (95% CI 0·32–0·42). Kufumanekisa ukuba inani labantu abafunywanwa yile ntsholongwane ayenyuka xa amajoni omzimba esihla okanye haemoglobin isihla, kwaye yenze bhetele kune lipoarabinomannan yomchamo kwaba ilactate ibiphezulu. Iziphumo zamanani ze Xpert Ultra egazini zibonise unxulumana nosweleka ngaphezulu kwezinye ivanvanyo njenge culture yegazi, lipoarabinomannan yomchamo, okanye Xpert yomchamo. iPrincipal component yobume besigulo ifumanise omaki be inflammation, omaki bomonakalo wenyama nomonakalo wamalungu omzimba bunxulumane nokufunyaniswa kwentsholongwane egazini nge Xpert

Ultra (nxulumana nokunyuka kwe standard deviation nge 1·1 kwi PC score, p<0·0001, kwaye nomda womjikelo (*r*= –0·5; p<0·0001).

Ukutolikwa kweziphumo: Uvavanyo lwe gazi elihluziwe nge Xpert Ultra lunga setyenziswa ukuxilonga intsholongwane yesifo sephepha ngoku khawulezileyo kwizigulana ezigula kakhulu ezikrokreleka isifo sephepha esinxulumene ne ntsholongwane ka gawulayo, kwaye inika ulwazi olungaphezulu ngenkqubo yobunkenenkene ngesifo xa kuthelekiswa nezinye imvavanyo. Ubudlelwane phakathi kwe dosi nokuphendulwa kwayo, nobungakani kweziphumo ze Xpert Ultra egazini kunye noku phendula komzimba kwingulo nomngcipheko wokufa kongeza ubungqina obucebisa ukuba ubuninzi be ntsholongwane i*M tuberculosis* esuleleke egazini buno budlelwane neziphumo zogula sisifo sephepha.
