# Supplementary material for: Xpert Ultra testing of blood in severe HIV-associated tuberculosis to detect and measure Mycobacterium tuberculosis blood stream infection: a diagnostic and disease biomarker cohort study
Source: Lancet Microbe. 2022 Jul;3(7):e521–32. doi: 10.1016/S2666-5247(22)00062-3 (PMC9242865; doi:10.1016/S2666-5247(22)00062-3)
Supplement: Afrikaans translation of the abstract [file mmc2.pdf]

THE LANCET
Microbe

Supplementary appendix 2

This translation in Afrikaans was submitted by the authors and we reproduce it as supplied. It has not been peer reviewed. *The Lancet*'s editorial processes have only been applied to the original in English, which should serve as reference for this manuscript.

Hierdie vertaling in Afrikaans is deur die outeurs voorgelê en ons gee dit weer soos dit aan ons verskaf is. Dit is nie eweknie-beoordeel nie. The Lancet se redaksionele prosesse is slegs op die oorspronklike Engelse teks toegepas, en laasgenoemde moet as bronverwysing vir hierdie manuskrip gebruik word.

Xpert Ultra toets van bloed in ernstige MIV-geassosieerde tuberkulose om *Mycobacterium tuberkulose* bloedstroom infeksie op te spoor en te meet: 'n diagnostiese en siekte biomerker kohort studie

Agtergrond: *Mycobacterium tuberkulose* bloedstroom infeksie is 'n leidende oorsaak van dood in mense wat met MIV leef en verspreide (gedissemineerde) bacillêre lading kan 'n belangrike drywer van siekte erns wees. Ons doel was om Xpert MTB/RIF Ultra (Xpert Ultra) toetsing van bloed te evalueer as 'n diagnostiese merker vir *M tuberkulose* bloedstroom infeksie en ondersoek in te stel na die Xpert toets se siklus drempelwaarde as 'n kwantitatiewe siekte biomerker.

Metodes: In hierdie kohortstudie het ons gestoorde bloedmonsters verkry van 'n groot en goed gekenmerkde kohort van volwasse pasiënte wat in die in Wes-Kaap, Suid-Afrika gehospitaliseer is met vermoedelik MIV-geassosieerde tuberkulose en 'n CD4-telling van minder as 350 selle per µL. Pasiënte wat reeds anti-tuberkulose terapie ontvang het, is uitgesluit. Monsters is verkry tydens werwing binne 72 h van toelating tot die hospitaal, en pasiënte is vir 12 weke opgevolg om uitkoms te bepaal. Ons het die gestoorde bloedmonsters getoets met die Xpert Ultra platform na prosessering van die bloed met lysis en was stappe. Ons het diagnostiese opbrengs (proposie gevalle opgespoor terwyl onbeskikbare toetsuitslae as negatief gedokumenteer word) teen 'n mikrobiologiese verwysing standaard beoordeel, beide as 'n funksie van merkers van kritieke siekte en in vergelyking met ander vinnige diagnostiese merkers (uriene lipoarabinomannan en sputum Xpert). Kwantitatiewe bloed Xpert Ultra resultate is geëvalueer as 'n siekte biomerker deur die evaluasie van die assosiasie met siekte fenotipe gedefinieer deur hoofkomponent analise van 32 gasheer-reaksie merkers. Prognostiese waarde in vergelyking met ander tuberkulose biomerkers is ge-evalueer met behulp van waarskynlikheidsverhoudingstoetsing van geneste modelle wat 12-week sterftes voorspel.

Bevindinge: Tussen 16 Januarie 2014 en 19 Oktober 2016 is 659 deelnemers gewerf in die kohort studie en 582 het 'n gestoorde bloedmonster beskikbaar gehad. 447 (77%) van 582 het aan die mikrobiologiese verwysingstandaard vir tuberkulose diagnose voldoen. Die mediaan CD4 telling was 62 (interkwartielvariasiewydte 221-33) selle per µL, en 123 (21%) van die deelnemers het gesterf gedurende die 12-weke opvolg periode. Bloed Xpert Ultra was positief in 165 (37%) van 447 deelnemers met bevestigde tuberkulose deur die uitgebreide mikrobiologiese verwysingsstandaard met 'n diagnostiese opbrengs van 0·37 (95% vertrouensinterval 0·32–0·42). Diagnostiese opbrengs het toegeneem met laer CD4-telling of hemoglobien, en beter presteer as uriene lipoarabinomannan toetsing in deelnemers met verhoogde veneuse laktaat. Kwantitatiewe bloed Xpert Ultra resultate was nouer geassosieer met mortaliteit as ander tuberkulose biomerkers, insluitend bloedkultuur, en uriene lipoarabinomannan, of uriene Xpert ($p<0·05$). 'n Hoofkomponent van kliniese fenotipe wat merkers van inflammasie, weefselskade en orgaandisfunksie insluit, is sterk geassosieer met

beide bloed Xpert-Ultra positiwiteit (wat verband hou met 'n standaard afwyking-toename van 1·1 in hoofkomponent telling, $p<0·0001$), en siklusdrempel waarde(r= -0·5; $p<0·0001$).

Interpretasie: Xpert Ultra-toetsing van voorafverwerkte bloed kan gebruik word as 'n vinnige diagnostiese toets in kritiese siek pasiënte met vermoedelike MIV-geassosieerde tuberkulose, terwyl dit ook addisionele prognostiese inligting gee in vergelyking met ander beskikbare merkers. 'n Dosis-verwante verhouding tussen kwantitatiewe bloed Xpert Ultra resultate, gasheer-reaksie fenotipe en mortaliteit risiko voeg by tot bewyse wat daarop dui dat M *tuberkulose* bloedstroom infeksie bacillêre lading oorsaaklik verwant is aan pasiënt uitkoms.
