# Supplementary material for: Xpert Ultra testing of blood in severe HIV-associated tuberculosis to detect and measure Mycobacterium tuberculosis blood stream infection: a diagnostic and disease biomarker cohort study
Source: Lancet Microbe. 2022 Jul;3(7):e521–32. doi: 10.1016/S2666-5247(22)00062-3 (PMC9242865; doi:10.1016/S2666-5247(22)00062-3)
Supplement: Supplementary appendix 3 [file mmc3.pdf]

THE LANCET
Microbe

Supplementary appendix 3

This appendix formed part of the original submission and has been peer reviewed. We post it as supplied by the authors.

Supplement to: Boloko L, Schutz C, Sibiya N, et al. Xpert Ultra testing of blood in severe HIV-associated tuberculosis to detect and measure *Mycobacterium tuberculosis* blood stream infection: a diagnostic and disease biomarker cohort study. *Lancet Microbe* 2022; published online May 26. https://doi.org/10.1016/S2666-5247(22)00062-3.

Supplemental appendix

For manuscript

Xpert Ultra testing of blood in severe HIV-associated tuberculosis to detect and measure M. tuberculosis blood stream infection: a diagnostic and disease biomarker study

Authors

Linda Boloko MMed, Charlotte Schutz PhD, Nomfundo Sibiya PhD, Avuyonke Balfour MSc, Amy Ward MBChB, Muki Shey PhD, Mark P Nicol PhD, Rosie Burton MBChB, Robert J. Wilkinson PhD, Gary Maartens MMed, Graeme Meintjes PhD, David A. Barr PhD

Contents

STARD checklist	Page 1 – 2
Supplemental methods	Page 4
Supplemental figure and tables	Page 6 – 14
Systematic review & meta-analysis for research in context box	Page 17

STARD checklist

Section & Topic	No	Item
**METHODS**		
Study design	**5**	Whether data collection was planned before the index test and reference standard were performed (prospective study) or after (retrospective study)
The data collection was planned and completed before the index test; the data collection was planned before the reference standard.		
Participants	**6**	Eligibility criteria
	**7**	On what basis potentially eligible participants were identified (such as symptoms, results from previous tests, inclusion in registry)
	**8**	Where and when potentially eligible participants were identified (setting, location and dates)
	**9**	Whether participants formed a consecutive, random or convenience series
Patients were recruited to the parent study if they were newly admitted to hospital (preceding 48hours), were HIV positive with CD4 count < 350 cells/mm3, and the admitting clinicians and study clinicians both suspected a new diagnosis of tuberculosis, i.e. patients who clinicians had a high index of suspicion for tuberculosis and who were being tested for tuberculosis. These patients were identified by screening all newly admitted patients in preceding 24 hours, on medical wards and emergency department, Monday to Friday mornings, with review of patient admission notes and test results. This occurred January 2013–October 2016 . Participants were consecutive, but if more patients were available for recruitment than could be managed due to staffing levels on a given day, participants were randomly selected from eligible patients. Patients who had missing biobanked blood samples due to these samples being lost or used in another research study were excluded (considered missing completely at random).		
Test methods	**10a**	Index test, in sufficient detail to allow replication
	**10b**	Reference standard, in sufficient detail to allow replication
	**11**	Rationale for choosing the reference standard (if alternatives exist)
Index test is described in para 3 of methods section main manuscript, and shown in supplementary video file. Reference tests are described in “laboratory procedures” subsection of methods section. Because no gold standard diagnostic for TB exists, and diagnostics perform less well in advanced HIV, we chose a reference standard utilising a large number of available diagnostics combined. We combined all available well validated diagnostics in a strict microbiological reference standard to assess sensitivity; we used an extended reference standard to assess diagnostic yield which included the index test and urine-LAM. The later is justified because it is plausible that the strict microbiological reference standard has imperfect sensitivity particularly for critically ill patients (and we aimed to capture diagnostic utility specifically in this group), and also because the molecular detection of MTB by PCR in blood (a sterile site) should in theory have high specificity in a patient population at very high risk of tuberculosis. The use of two reference standards is thus a purposeful response to the absence of a gold standard.		
	**12a**	Definition of and rationale for test positivity cut-offs or result categories of the index test, distinguishing pre-specified from exploratory
	**12b**	Definition of and rationale for test positivity cut-offs or result categories of the reference standard, distinguishing pre-specified from exploratory
No de novo cut-offs were used (the inbuilt cycle threshold cut-off intrinsic to the Xpert-Ultra cartridge software and the manufacturer recommended cut-off for urine-LAM testing were used)		
	**13a**	Whether clinical information and reference standard results were available to the performers/readers of the index test
	**13b**	Whether clinical information and index test results were available to the assessors of the reference standard
Performers of the index test were blinded to clinical and reference standard data. The analysis was performed by authors with access to the full dataset including these variables.		
Analysis	**14**	Methods for estimating or comparing measures of diagnostic accuracy
	**15**	How indeterminate index test or reference standard results were handled
	**16**	How missing data on the index test and reference standard were handled
Sensitivity, specificity, and diagnostic yield defined in “Diagnostic utility analysis” subsection of methods section. For sensitivity, indeterminant results and results missing due to non-obtainment of sample were removed from the numerator and denominator. For diagnostic yield, indeterminant results and results missing due to non-obtainment of sample were coded as “negative” and included in		

numerator and denominator. This allows the differential difficulty in obtaining some samples to be incorporated in the later measure of diagnostic utility.		
	**17**	Any analyses of variability in diagnostic accuracy, distinguishing pre-specified from exploratory
Analysis of variation in diagnostic yield by patient characteristics was a primary aim of the study and prespecified for patient covariates CD4 count, haemoglobin, lactate and survival, as detailed in "Diagnostic utility analysis" subsection of methods section.		
	**18**	Intended sample size and how it was determined
Sample size was limited by availability of samples from parent study but was determined to give >90% power to detect a 10% absolute difference in diagnostic yield at the 0.95 confidence level comparing two diagnostics by binomial distribution.		
**RESULTS**		
Participants	**19**	Flow of participants, using a diagram
Figure S3		
	**20**	Baseline demographic and clinical characteristics of participants
Table 1		
	**21a**	Distribution of severity of disease in those with the target condition
Test performance is described as a function of disease severity markers as a primary aim of analysis: Figure 1 and table 2		
	**21b**	Distribution of alternative diagnoses in those without the target condition
Table 1 B		
	**22**	Time interval and any clinical interventions between index test and reference standard
Index and reference standard samples were collected at same timepoint. Index tests were performed on biobanked samples after end of clinical recruitment.		
Test results	**23**	Cross tabulation of the index test results (or their distribution) by the results of the reference standard
Figure 1 and table 2 main manuscript		
	**24**	Estimates of diagnostic accuracy and their precision (such as 95% confidence intervals)
Figure 1 and table 2 main manuscript		
	**25**	Any adverse events from performing the index test or the reference standard
Index test performed after end of study so no ability to negatively influence patient care. There were no occurrences of significant adverse events related to venesection in the parent study.		

Supplementary methods

Inclusion & exclusion criteria in the parent study

Inclusion criteria	Exclusion criteria
1. HIV-associated tuberculosis suspected as a cause of admission to hospital by admission clinicians (recorded on differential diagnosis).<br>2. 18 years or older<br>3. Informed consent from patient (unless drowsy/confused)<br>4. HIV seropositive<br>5. CD4 count ≤ 350cells/mm3	1. HIV seronegative or testing declined<br>2. Pregnant<br>3. 3 or more doses of TB treatement received during the admission or has been on TB treatment within one month of admission

Clinical and immunological variables assessed for association with blood Xpert-Ultra

Immunological variables	Clinical variables
IL1ra, IL-4, IL-5, IL-6, IL-7, IL-8, IL-12p70, IL-13, IL-17, MIPa, MIPb, FGF, PDGF, RANTES, TGF-b1, IP10	CRP, procalcitonin, lactate, albumin, ALT, bilirubin, AST, haemoglobin, CD4, lymphocyte, monocyte, neutrophil, D-dimer, platelets, creatinine, bicarbonate

ALT: alanine transferase, AST: aspartate transferase, CRP: C-reactive protein

Bacterial blood culture SOP

For non-mycobacterial blood stream infection pathogen detection, bacterial blood cultures (5-10mL blood in BacT/ALERT® Culture Media) were performed by the parent study if the patient had not received antibiotics at the time of enrolment, and also by clinical teams managing patients when bacterial BSI was suspected. Automated detection of growth in BacT/ALERT® Microbial Detection System was further assessed for pathogen identification using the Groote Schuur microbiology department SOP. In brief, secondary cultures on selective media directed by Gram staining were performed (including but not limited to 2% bile aesculin, MacConkey, blood agar), use of biochemical testing, and use of Vitek 2 GN-ID and GP-ID cards as required.

Blood Xpert Ultra testing demonstrative video

https://vimeo.com/413202354

Supplementary figures and tables

Index:

Figure S1: **Pre-clinical blood pre-processing method development**
Figure S2. **Imputation model for “trace” positive blood Xpert-ultra samples**
Figure S3. **Inclusion flow chart**
Figure S4. **Qualitative & quantitative associations between tuberculosis detection modalities**
Figure S5. **32 variables association with blood Xpert Ultra positivity & Ct value (four sets of 16 panels).**
Figure S6. **Association between blood Xpert-ultra on ordinal scale and 32 clinical/immunological markers**
Figure S7: **Indirect comparison of blood Xpert-Ultra sensitivity in TB blood culture positive patients by sample storage condition.**

Table S1. **Clinical characteristics of patients who had samples available and those not available for blood Xpert Ultra**

Table S2. **Two patients with positive blood Xpert Ultra but no other confirmatory test positive for tuberculosis.**

Figure S1: **Pre-clinical blood pre-processing method development**

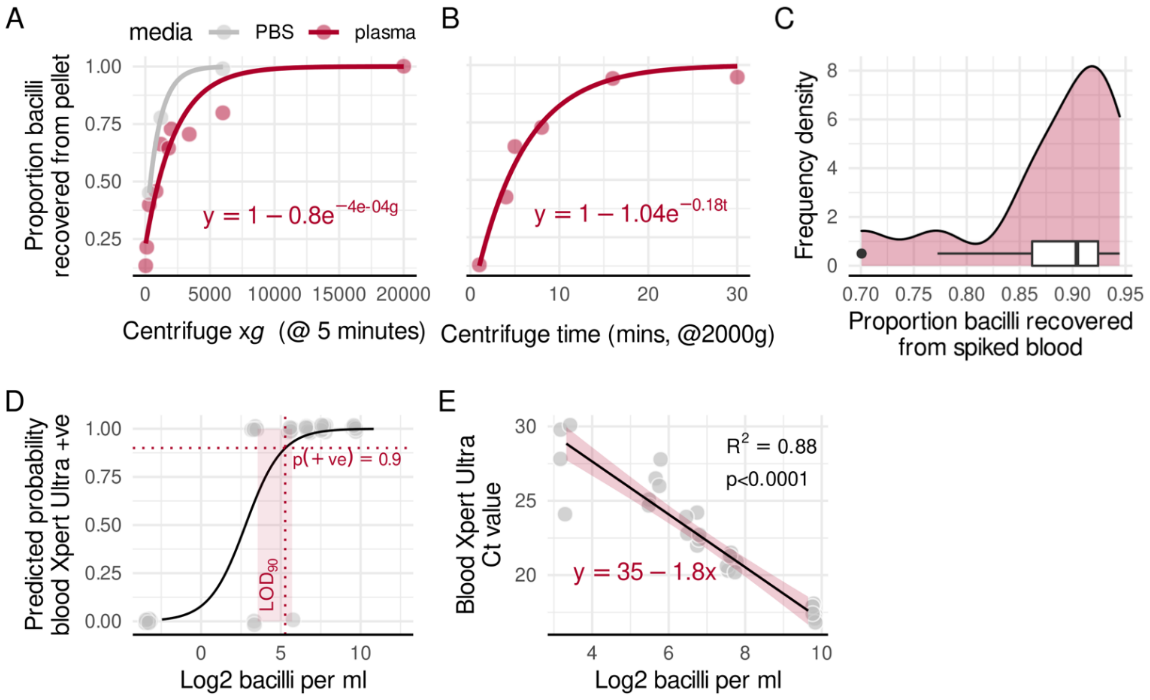

Proportion of bacilli recovered after pelleting spiked plasma, as a function of centrifuge x*g* (**A**) and time (**B**). Number of bacilli in mid-log broth cultures of *M. bovis* BCG were quantified using a flow cytometry method for absolute counting of mycobacteria.[https://doi.org/10.1101/2021.05.01.442251] This method is more accurate than colony forming unit counting, because, unlike CFU counting, the flow cytometry method observes intact non-colony forming bacilli and bacilli aggregates (clumps). A known number of bacilli were then spiked into 1.5ml aliquots of a plasma derived from heparinised healthy volunteer blood samples. These were made up to a volume of 3ml by addition of 1.5ml PBS, and then centrifuged using different centrifuge parameters shown, with subsequent removal of 90% supernatant and resuspension to starting volume by pipetting. Bacilli in these processed samples were then enumerated using the flow cytometry method and compared to a control which did not undergo centrifugation. Three samples suspended in PBS (no plasma) were also processed as a comparator.

Proportion of bacilli recovered from spiked healthy volunteer blood after blood processed by full lysis-wash SOP used for the Xpert Ultra method (**C**). *M. bovis* BCG bacilli grown and quantified as above were heat treated and stained with SYBR-gold (ThermoFisher, S-11494) before being spiked at known concentration into 3ml samples of healthy volunteer blood. Blood samples (n=12) were then processed using the lysis-wash protocol described in main manuscript for pre-processing blood for Xpert-Ultra testing. The number of bacilli in the resulting lysate were quantified using fluorescence microscopy amd compared to the original number spiked to measure the proportion recovered after any losses incurred from the blood lysis-wash protocol.

Estimating blood Xpert Ultra protocol limit of detection (LOD, **D**) and relationship between number of bacilli present and Ct value (**E**) using spiked healthy volunteer blood samples. A fully attenuated *M. tuberculosis* strain with auxotrophic mutations in leucine and pantothenate biosynthesis was grown and quantified using the flow cytometry method, before being spiked at varying concentrations (0, 10, 50, 100, 200, 800 bacilli per ml, each 3-6 reps) into 3ml aliquots of heparinised healthy volunteer blood. These samples were then processed using the blood Xpert Ultra protocol described in main manuscript (omitting freeze-thaw step). To estimate LOD where 90% of samples are expected to be positive, a logistic regression model was fit (D, black sigmoid curve) onto the raw data (D, grey points, where positive = 1 and negative = 0). The value of bacilli per ml with 90% predicted probability for detection by blood Xpert Ultra (D, intersection of dashed lines) was then estimated (= 40 bacilli per ml). 50%CI (30 to 50 bacilli per ml) for this estimate were generated by 1000-fold bootstrapping (D, shaded area). For spiked blood that was positive by Xpert Ultra testing, summary Ct values were extracted as per the method described in main manuscript, and compared to spiked bacilli per ml on log_2 scale by linear regression (E, the evidence of higher variance at lower spiked bacilli counts has not been modelled). A doubling in bacilli count is estimated to result in a fall in Ct value of ~1.8, a slightly steeper fall than might have been expected.

Figure S2. **Imputation model for "trace" positive blood Xpert Ultra samples**

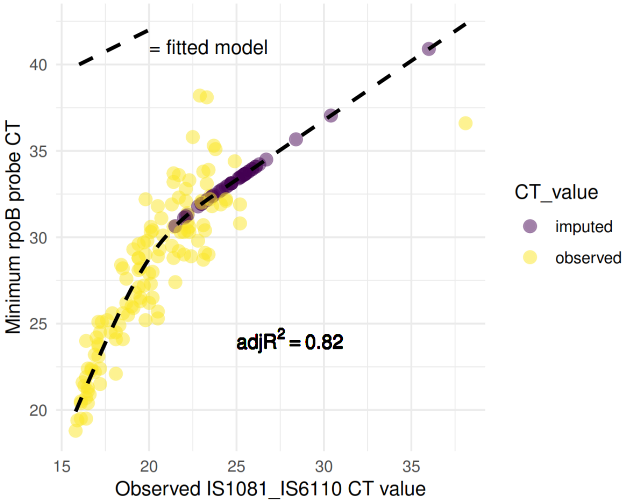

A restricted cubic spline model with 3 knots regressed observed minimum *rpoB* probe Ct values on observed IS1081-IS6110 probe Ct values (dashed black line fit to yellow points), and had adjusted R^2 0.82. The model was used to impute missing minimum *rpoB* probe Ct values from observed IS1081-IS6110 probe Ct values in "trace" positive samples.

Figure S3. **Inclusion flow chart**

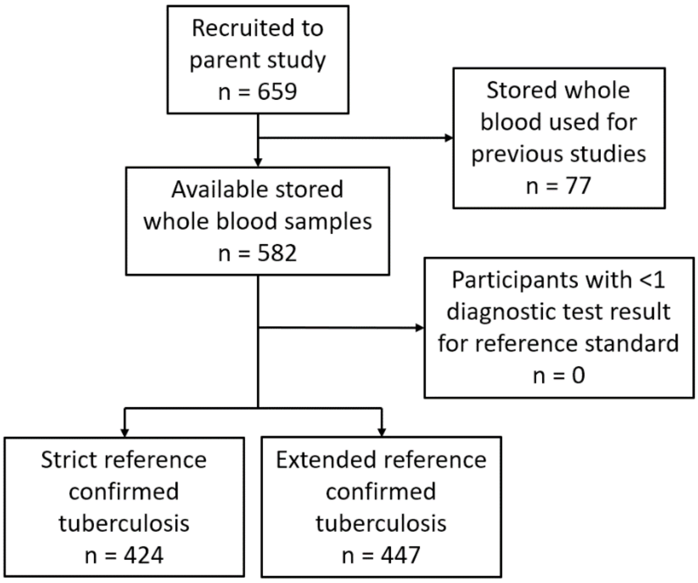

Figure S4 **Qualitative & quantitative associations between tuberculosis detection modalities**

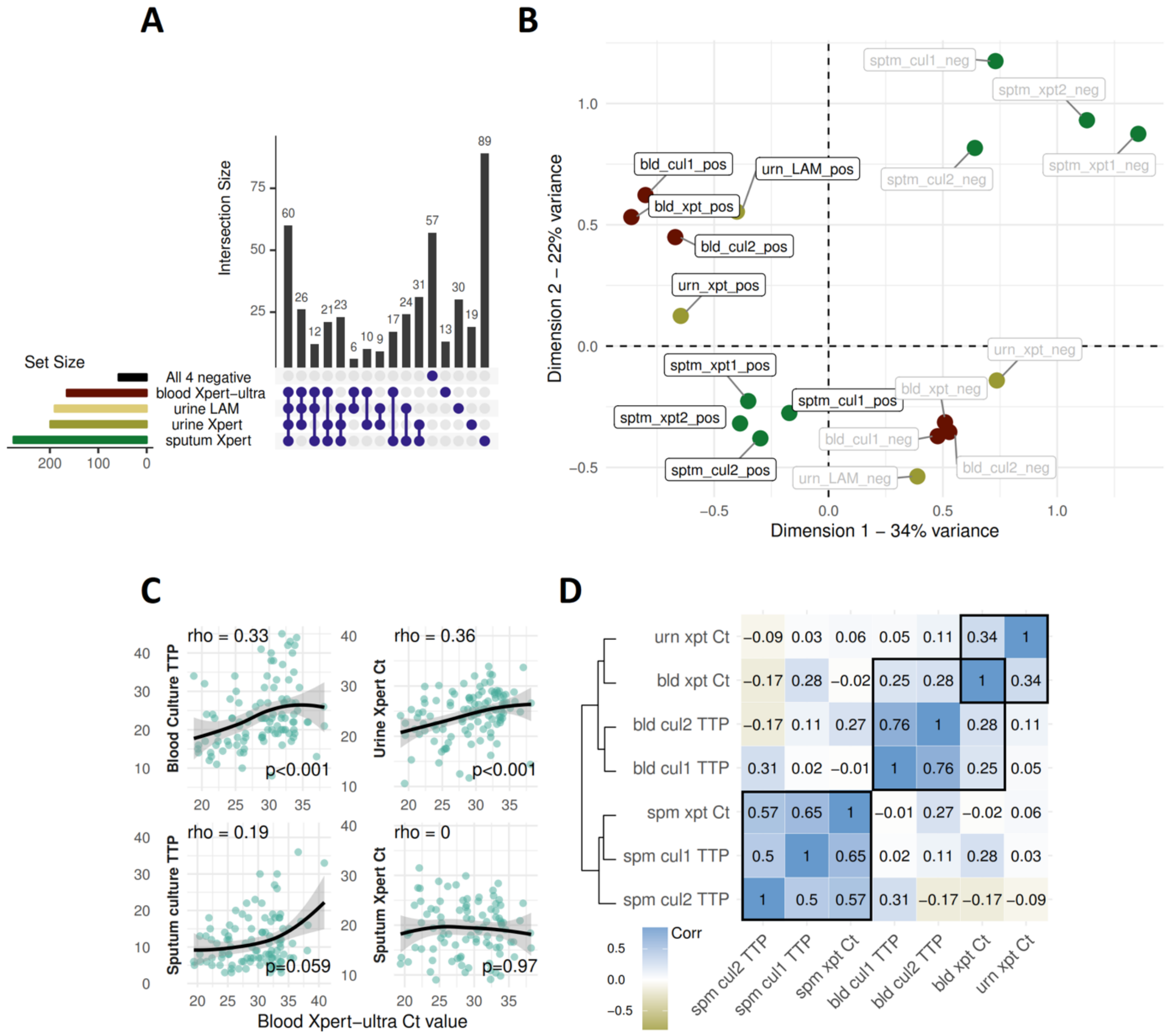

	spm cul2 TTP	spm cul1 TTP	spm xpt Ct	bld cul1 TTP	bld cul2 TTP	bld xpt Ct	urn xpt Ct
urn xpt Ct	−0.09	0.03	0.06	0.05	0.11	0.34	1
bld xpt Ct	−0.17	0.28	−0.02	0.25	0.28	1	0.34
bld cul2 TTP	−0.17	0.11	0.27	0.76	1	0.28	0.11
bld cul1 TTP	0.31	0.02	−0.01	1	0.76	0.25	0.05
spm xpt Ct	0.57	0.65	1	−0.01	0.27	−0.02	0.06
spm cul1 TTP	0.5	1	0.65	0.02	0.11	0.28	0.03
spm cul2 TTP	1	0.5	0.57	0.31	−0.17	−0.17	−0.09

A. Intersections between sets defined by positive rapid-test results. Total set size (number of patients with positive sputum Xpert, positive urine Xpert, positive blood Xpert Ultra, and number of patients with all 4 tests negative) shown by horizontal coloured bars. Intersections of these sets are indicated by the connected blue dots; number of patients in each of the possible intersections are shown with vertical bars. These figures are based on a single test result as described in main text. **B**. Factor analysis describing the main dimensions of variation seen for qualitative test results (positive or negative) including where two samples were sent for the same test. The first dimension of variation (capturing 34% of total variance in test results between patients) separates patients with predominantly positive and negative test results; the second dimension of variation (explaining 22% variance) separates patients by compartment yielding positive results, with blood and urine diagnostics separating from sputum based diagnostics. Categories further from the origin are less frequent (higher variance) than those near the origin. **C**. Correlation between quantitative read-outs from tests (sputum and blood culture time to positivity, TTP; urine and sputum Xpert Ct values) and

blood Xpert Ultra Ct value. Fitted line and shaded 95% confidence interval from LOESS smoothing function; Rho and p value from Spearman's rank test. **D**. Pearson's correlations between time to positivity of cultures (TTP) and Ct values from blood, urine and sputum (bld, urn, spm) samples, ordered using hierarchical clustering.

Figure S5. **32 variables association with blood Xpert Ultra positivity & Ct value (four sets of 16 panels).**

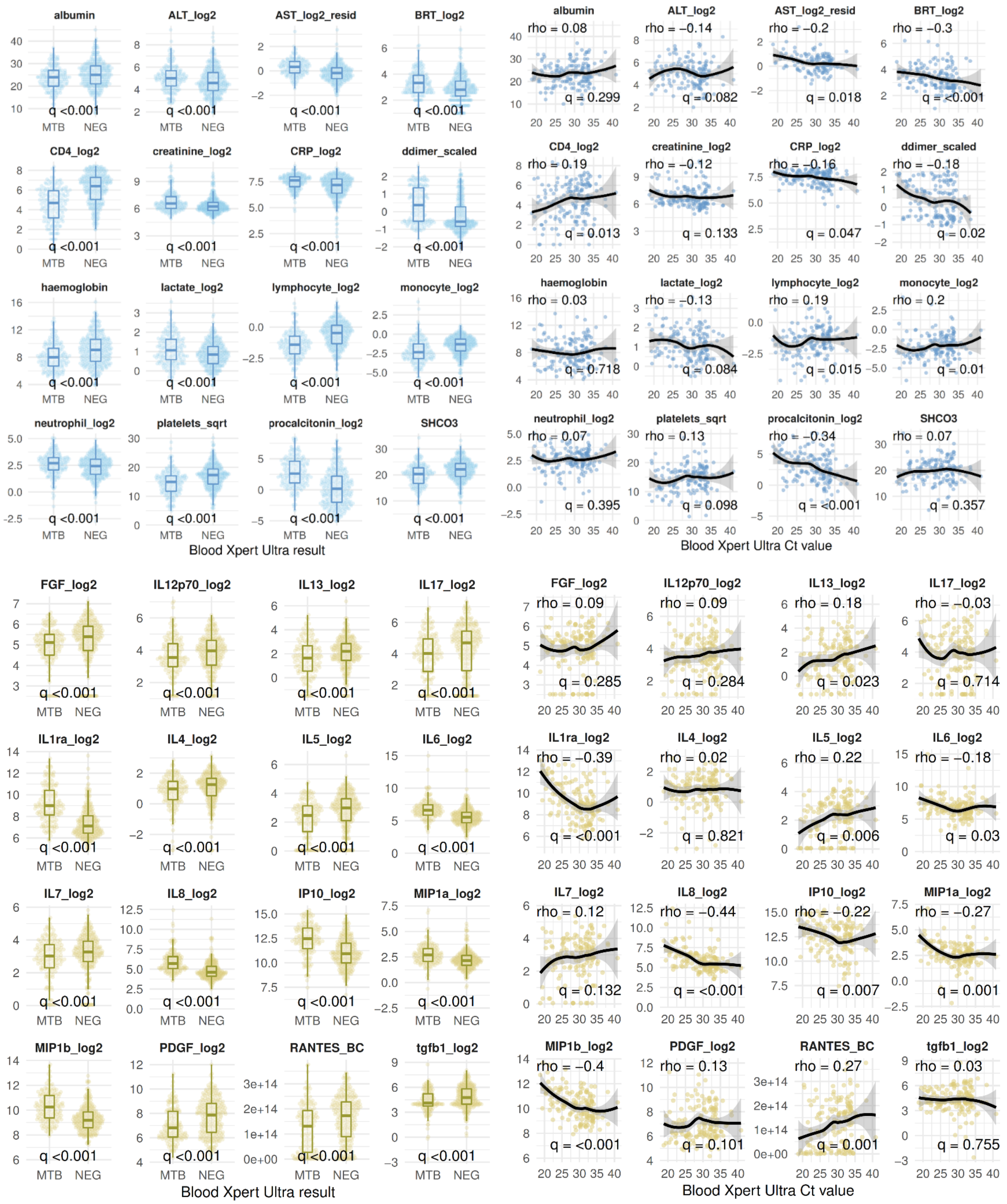

Left panels show results of blood assays (y-axis) by blood Xpert Ultra result (x-axis; MTB = *M. tuberculosis* detected at any level, NEG = negative): points are arranged in 'violin scatterplots' showing the density distribution for y-axis variable; overlaid box-plots show median, interquartile range, and range. Benjamini-Hochberg corrected q-values from t-tests are shown. **Right panels** show scatter plots for blood Xpert Ultra

Ct values (x-axis) versus blood assay results (y-axis) for the subset of patients with a positive blood Xpert Ultra result. A smoothed, non-parametric regression line with 95% confidence interval (shaded band) for the fit is overlaid. Spearman's rank correlation (Rho) is shown along with associated Benjamini-Hochberg corrected q-values for the rank correlation.

Variables were transformed to be approximately normal using log to base 2 (log2), square-root (sqrt), or box-cox (BC) transformations as indicated by suffix. D-dimer values for the cohort were from 2 assays measured on different scales; these were combined by standardising both sets of observed values to have mean 0 and sd 1 (ddimer_scaled variable).

ALT = alanine aminotransferase; AST resid = residual variation in asparate transaminase independent of covariance with ALT; BRT = total bilirubin; CD4 = CD4 cell count; CRP = C-reactive protein; lymphocyte, monocyte, neutrophil & platelet variables = peripheral blood counts of these variables; SHCO3 = venous standard bicarbonate; IL = interleukin; IL1ra = interleukin-1 receptor antagonist; FGF = basic fibroblast growth factor; IP10 = interferon gamma-induced protein; MIP1a = macrophage inflammatory protein-1 alpha; MIP1b = macrophage inflammatory protein-1 beta; PDGF = platelet-derived growth factor-BB (PDGF); RANTES = regulated on activation, normal T cell expressed and secreted; tgfb1 = transforming growth factor beta-1.

Figure S6. **Association between blood Xpert-ultra on ordinal scale and 32 clinical/immunological markers**

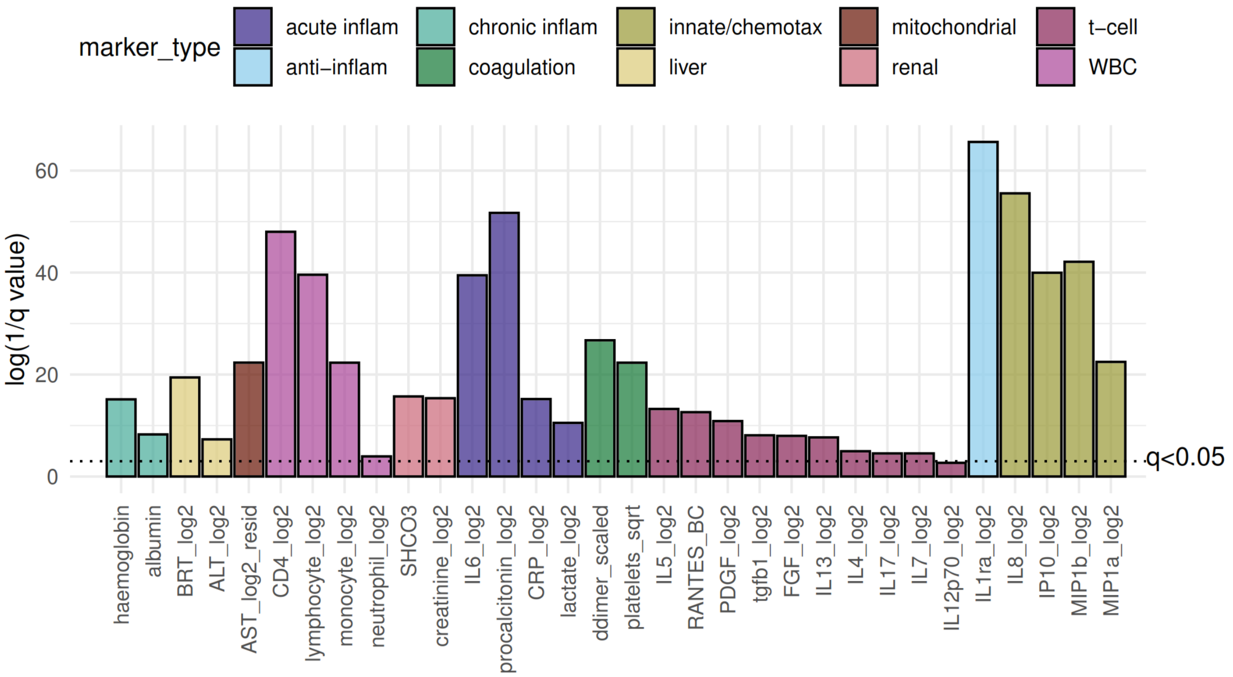

Manhattan plots showing strength of association between 16 clinical and 16 immunological variables (same as figure S3) and blood Xpert-ultra result represented on an ordinal scale where negative test = 0 and levels 1-3 are defined by tertiles of Ct values from positive tests. P-values from Spearman's rank correlation test corrected for multiple comparison using Benjamini-Hochberg procedure to limit false discovery rate, indicated by q-values. Horizontal dashed line = q-value 0·05; variables above this line are significant at the 0·05 level after correction for multiple comparisons. ALT = alanine aminotransferase; AST resid = residual variation in asparate transaminase independent of covariance with ALT; BRT = total bilirubin; CD4 = CD4 cell count; CRP = C-reactive protein; lymphocyte, monocyte, neutrophil & platelet variables = peripheral blood counts of these variables; SHCO3 = venous standard bicarbonate; IL = interleukin; IL1ra = interleukin-1 receptor antagonist; FGF = basic fibroblast growth factor; IP10 = interferon gamma-induced protein; MIP1a = macrophage inflammatory protein-1 alpha; MIP1b = macrophage inflammatory protein-1 beta; PDGF = platelet-derived growth factor-BB (PDGF); RANTES = regulated on activation, normal T cell expressed and secreted; tgfb1= transforming growth factor beta-1

Figure S7: **Indirect comparison of blood Xpert-Ultra sensitivity in TB blood culture positive patients by sample storage condition.**

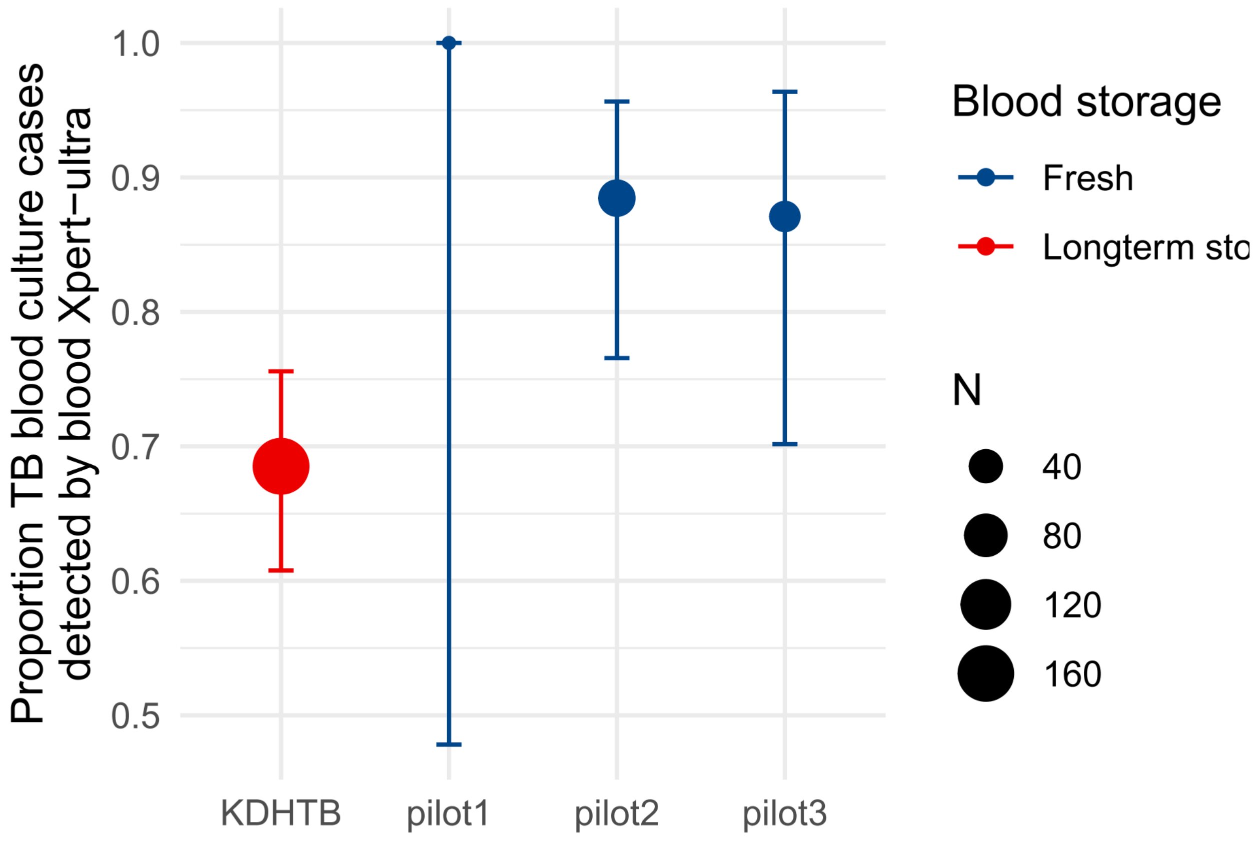

Post-hoc analysis in response to reviewer request. In the study presented in the manuscript, biobanked blood samples stored at -80oC were used for blood Xpert Ultra testing ("Longterm storage -80ºC"), which may have impacted on sensitivity of detection of M.tb. Three pilot studies separate from the main study were performed using blood processed for Xpert Ultra directed without storage, or in some cases blood stored for up to 14 days at -20ºC for batch processing ("Fresh"). Here the proportion of MTBBSI samples (defined by having a positive TB blood culture from concurrent blood sample) detected by a single blood Xpert Ultra test are compared across the studies. All the pilots using "fresh" blood for Xpert Ultra had higher apparent sensitivity for detecting MTBBSI. 95% CI for the proportion detected are indicated with error bars for each pilot and the main study. In a Bayesian mixed-effects binomial regression model allowing random effects by study on this data, posterior probability of lower sensitivity from longterm storage of blood was estimated to be 90%. The model predicts that on average fresh samples will have +20% absolute higher sensitivity compared to stored samples (95%CrI -17 to +79% difference).

Table S1. **Clinical characteristics of patients who had samples available and those not available for blood Xpert Ultra**

Characteristic	Sample available for blood Xpert Ultra, N = 582[1]	No sample available for blood Xpert Ultra, N = 77[1]	p value[2]
**Age, years**	36 (31, 44)	35 (31, 44)	0.7
**Sex,** Female	303 (52%)	39 (51%)	0.8
**CD4 count, cells/mm3**	62 (22, 132)	50 (22, 93)	0.2
**ART status**			0.06
Defaulted	133 (23%)	26 (34%)	
Naive	220 (38%)	30 (39%)	
On ART	222 (39%)	21 (27%)	
**Cough**	386 (69%)	58 (82%)	0.03
**Loss Of Appetite**	369 (66%)	43 (59%)	0.2
**Loss Of Weight**	497 (90%)	69 (93%)	0.3
**Night Sweats**	307 (56%)	39 (54%)	0.8
**Heart rate, /min**	104 (94, 120)	102 (98, 118)	>0.9
**Temperature, °C**	36.70 (36.10, 37.50)	36.60 (36.00, 37.30)	0.4
**Systolic blood pressure, mmHg**	107 (97, 118)	112 (101, 120)	0.03
**Respiratory rate, /min**	20.0 (18.0, 24.0)	22.0 (20.0, 24.2)	0.12
**Venous lactate, mmol/L**	1.80 (1.30, 2.50)	1.90 (1.20, 2.80)	0.7
**Serum creatinine, µmol/L**	79 (59, 121)	80 (65, 142)	0.3
**CRP, mg/L**	154 (87, 232)	147 (104, 243)	0.8
**Sodium, mmol/L**	129.0 (125.0, 132.0)	129.0 (125.0, 132.0)	>0.9
**Haemoglobin, g/dL**	8.80 (7.23, 10.50)	8.90 (7.10, 11.00)	0.7
**White cell count, 10^9/L**	7.2 (4.6, 10.7)	6.2 (4.0, 8.8)	0.11
**Platelets, 10^9/L**	270 (178, 356)	250 (172, 330)	0.3
**Outcome at 12 weeks**			0.13
Died	123 (21%)	23 (30%)	
Loss to follow-up	12 (2.1%)	0 (0%)	

Survived	447 (77%)	54 (70%)

[1]Median (IQR); n (%)

[2]Wilcoxon rank sum test; Pearson's Chi-squared test; Fisher's exact test

Table S2. **Two patients with positive blood Xpert Ultra but no other confirmatory test positive for tuberculosis.**

Patient	Presenting complaints	CD4 count	Chest X-ray	Abdominal ultrasound	Management & outcome
1	Weight loss, night sweats, fever, diarrhoea.	31	Miliary infiltrates	Adenopathy, splenic microabscesses, renal collection	Treated for TB, and pyelonephritis without positive urine or blood culture, died 6 days into admission.
2	Cough, weight loss, night sweats, fever, diarrhoea.	10	Right pleural effusion	“Shotty” lymph nodes, enlarged right kidney	Treated for TB with documented improvement at 12 weeks, no alternative diagnosis made.

NAAT for tuberculosis detection in blood: systematic reviews and meta-analyses for Research in context box, Lancet Microbe submission

Linda Boloko, David Barr

13/12/2020

Contents

1 Preface **2**

2 Systematic Review 1: TB diagnosis using blood NAAT **2**
2.1 Introduction . . . 2
2.1.1 Aims & objectives . . . 2
2.1.2 Inclusions & exclusions . . . 2
2.1.3 Data for extraction . . . 2
2.1.4 Analysis plan . . . 2
2.1.5 Bias assessment . . . 3
2.2 Search strategies . . . 3
2.2.1 PubMed . . . 3
2.2.2 Scopus . . . 4
2.2.3 Combining search results, removing duplicates . . . 4
2.3 Screening results . . . 4
2.4 Description of included studies . . . 5
2.5 Descriptive summaries of reported diagnostic performance . . . 5
2.6 Bivariate regression modelling . . . 10
2.6.1 Meta-regression: covariates versus diagnostic performance . . . 10
2.7 Summary review 1 . . . 13

3 Systematic Review 2: *M. tuberculosis* blood stream infection diagnosis using blood NAAT **13**
3.1 Introduction . . . 13
3.1.1 Aims and objectives . . . 13
3.1.2 Included studies . . . 13
3.1.3 Data for extraction . . . 14
3.1.4 Analysis plan . . . 14
3.2 Screening results . . . 14
3.3 Comparing sensitivity of blood TB-NAAT and TB blood culture against an external reference standard . . . 14
3.4 Assessing sensitivity of blood TB-NAAT against TB blood culture as the reference standard . 16
3.5 Summary review 2 . . . 17

1 Preface

This document reports two systematic reviews and meta-analyses of literature on use of nucleic acid amplification technology (NAAT) to detect *M. tuberculosis* in patient blood samples. The first meta-analysis is focused on blood NAAT for TB diagnosis; the second is on bloood NAAT to diagnose *M. tuberculosis* blood stream infection (MTBBSI).

The motivation for these reviews is to inform the "Evidence before this study" section of the *Research in context* box for the KDHTB blood Xpert-ultra manuscript submission to *Lancet Microbe*.

This document is an Rmarkdown knitted as a pdf. This means alll the code for the analysis from raw data to final figures is embedded (so 100% reproducible). The code chunks are suppressed for readability but available at github repository.

2 Systematic Review 1: TB diagnosis using blood NAAT

2.1 Introduction

2.1.1 Aims & objectives

We want to systematically review literature on use of nucleic acid amplification technology (NAAT) to identify *M. tuberculosis* in patients' blood samples as a diagnostic for tuberculosis. Aims are to summarise:

1. What NAAT methods including blood pre-processing have been used for identifying *M. tuberculosis* in blood.
2. Reported sensitivity and specificity of blood *M. tuberculosis* NAAT **for tuberculosis**.

Systematic review and meta-analysis of studies using NAAT on blood to diagnose TB will be performed.

2.1.2 Inclusions & exclusions

Studies in which investigators used NAAT to identify *M. tuberculosis* in peripheral blood samples (whole blood or component) from patients identified prospectively with either suspected tuberculosis (e.g. cohort design) or confirmed tuberculosis diagnosis (e.g. case-control design) will be included.

Studies where it is unclear if patients were identified prospectively for blood NAAT testing (e.g. studies where inclusion was based on opportunistic receipt of a blood sample), studies where it is unclear what reference standard for tuberculosis diagnosis was, and studies reporting artificially spiked sample experiments (patient samples spiked with *M. tuberculosis ex vivo*) will be excluded.

2.1.3 Data for extraction

We will extract data on patient populations (adult versus paediatic, HIV status, TB prevalence, pulmonary versus extra-pulmonary, inpatient versus outpatient) and NAAT method (commercial v in-house, blood pre-processing, blood volume).

2.1.4 Analysis plan

Descriptive summaries using figures and tables. Bivariate random-effects regression acounting for correlation between sensitivity and specificity will be used to summarised central tendancies and heterogeneity. These will be fit using a Baysian (MCMC) approach implemented with the package *brms* in R studio. Meta-regression

on selected covariates will be performed using bivariate regression to test association between study and method covariates and diagnostic performance.

2.1.5 Bias assessment

Identified studies will be assessed for risk of bias using questions adapted from the QUADRA-2 tool:

- **Patient selection** . Are methods of patient selection adequately described (prior testing, presentation, intended use of index test and setting)? Was a consecutive or random sample of patients enrolled in a cohort design? Was HIV status of patients ascertained?
- **Index test** . Were the index test results interpreted without knowledge of the results of the reference standard?
- **Reference standard** . Were the reference standard results interpreted without knowledge of the results of the index test? Did all patients have at least 2 TB diagnostic tests (e.g. culture, NAAT, antigent testing, excluding the index test) performed from at least 2 different body sites (e.g. sputum and urine)? Was the index test excluded from the reference standard?
- **Flow and timing** . If blood NAAT was performed on only a subgroup of patients are the inclusion/exclusion criteria for this subgroup given? Was blood NAAT performed from samples taken at same timepoint as reference standard samples?

If answers to >1 or >3 of these questions are "no" or "unclear" risk of bias will be rated as moderate or high.

2.2 Search strategies

2.2.1 PubMed

Terms used in main PubMed search engine:

tuberculosis AND (blood OR mycobacteraemia OR "blood stream infection" OR bacteraemia OR bacillaemia) AND (NAAT OR PCR OR Xpert) AND diagnosis

This is translated by PubMed algorithm into an expanded search query which we have edited to remove irrelevant search terms (e.g. "blood" is linked to a range of haematology terms which are irrelavant and these have been removed by editing in the advanced search editor). This give a final expanded search query of:

("tuberculosi"[All Fields] OR "tuberculosis"[MeSH Terms] OR "tuberculosis"[All Fields] OR "tuberculoses"[All Fields] OR "tuberculosis s"[All Fields])
AND
("blood"[MeSH Subheading] OR "blood"[All Fields] OR "blood"[MeSH Terms] OR "mycobacteraemia"[All Fields] OR "blood stream infection"[All Fields] OR ("bacteraemia"[All Fields] OR "bacteremia"[MeSH Terms] OR "bacteremia"[All Fields] OR "bacteraemias"[All Fields] OR "bacteremias"[All Fields]) OR "bacillaemia"[All Fields])
AND
("nucleic acid amplification techniques"[MeSH Terms] OR ("nucleic"[All Fields] AND "acid"[All Fields] AND "amplification"[All Fields] AND "techniques"[All Fields]) OR "nucleic acid amplification techniques"[All Fields] OR "naat"[All Fields] OR "PCR"[All Fields] OR "Xpert"[All Fields]) AND ("diagnosable"[All Fields] OR "diagnosi"[All Fields] OR "diagnosis"[MeSH Terms] OR "diagnosis"[All Fields] OR "diagnose"[All Fields] OR "diagnosed"[All Fields] OR "diagnoses"[All Fields] OR "diagnosing"[All Fields] OR "diagnosis"[MeSH Subheading])

This retured 951 results which have been exported as a .nbib file (from above URL, [send to]–>[Citations Manager]–>[all results]–>[create file]) saved as *pubmed.nbib* in the working directory.

2.2.2 Scopus

Scopus was searched with query:

(TITLE-ABS-KEY (tuberculosis AND diagnosis) AND TITLE-ABS-KEY (pcr OR naat OR xpert) AND TITLE-ABS-KEY (blood OR mycobacteraemia OR bacteraemia OR bacillaemia OR "blood steam infection"))

Returning 537 results on 12/12/2020; these are exported as a .bib file using [select all–>BibTeX export, including abstract] saved as scopus.bib in the working directory.

2.2.3 Combining search results, removing duplicates

The .bib files are read in and combined, with duplicates identified by doi and removed.

2.3 Screening results

Summary of identified, screened, eligible and included study shown in PRISMA flow diagram in figure 1.

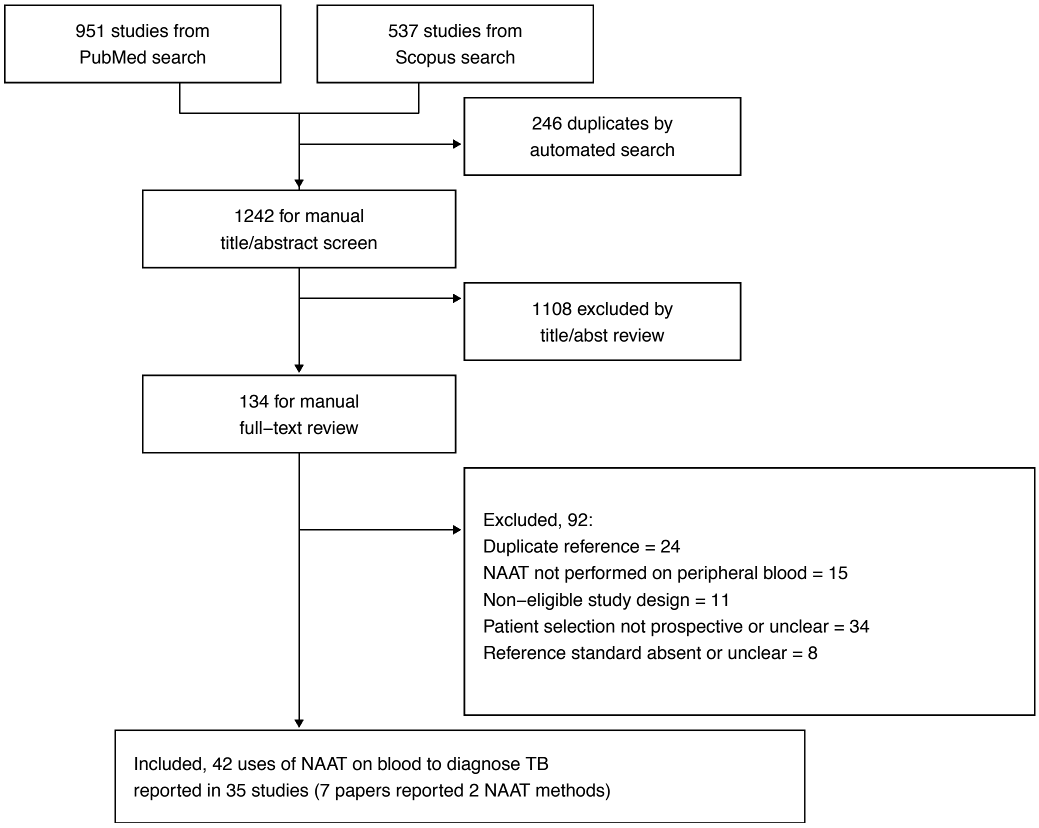

Figure 1: PRISMA flow chart for systematic review 1

2.4 Description of included studies

Characteristics of review 1 included studies are shown in figure 2 (study design, setting, patient population/cases, assessed risk of bias) and figure 3 (NAAT methods). A wide variety of study designs, patient populations and NAAT methods have been reported, with little replication of specific approaches. Initial reports of blood NAAT for TB diagnosis had a peak in the 1990s then a relative hiatus, followed by an increase again in last 10 years.

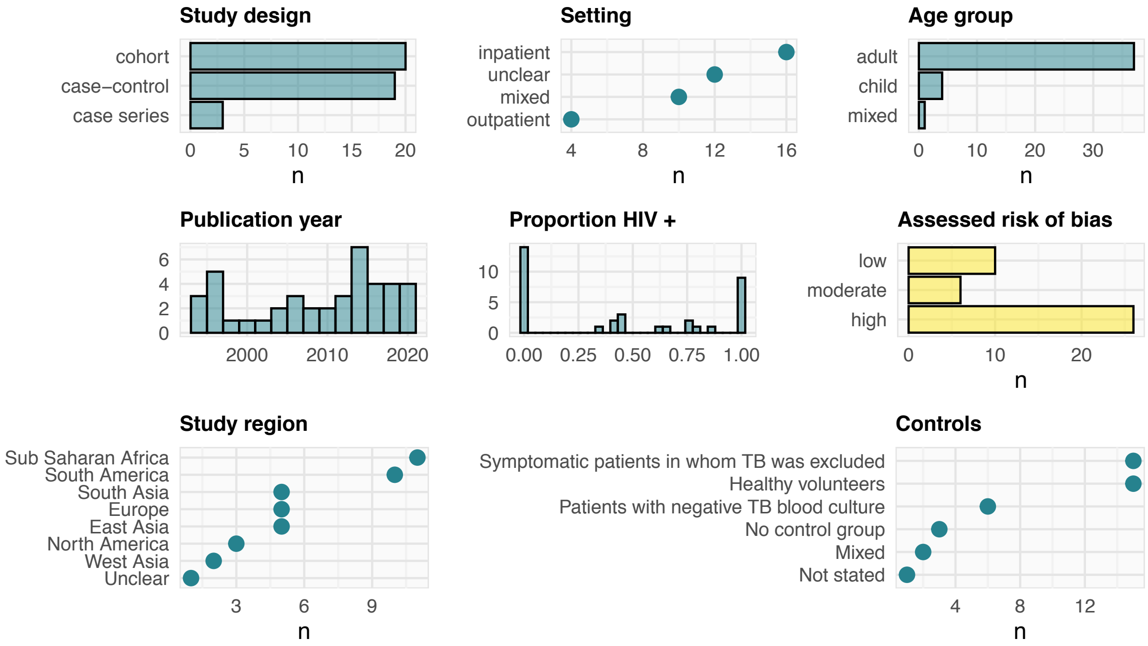

Figure 2: Description of review 1 included studies: study design, setting, patient population/cases, assessed risk of bias.

2.5 Descriptive summaries of reported diagnostic performance

Reported diagnostic performance in included studies are shown in figure 4-6. There are several striking findings. First, there is very marked heterogeneity in sensitivity (ranging from 0 to 100%), with very little evidence of correlation between sensitivity and specificity. Heterogeneity in sensitivity seems unrelated to study design, setting, HIV prevalance in study, or NAAT methods (blood volume, method of blood preprocessng) other than the finding that the studies using Xpert Rif/MTB had amongst the lowest reported sensitivities. Average reported sensitivity of blood NAAT seems to have slightly decreased over time since initial reports in 1990s.

However, reported sensitivity does appear to vary somewhat by assessed risk of bias, with studies assessed as lower risk of bias, and larger studies, reporting lower sensitivity.

There is also evidence of reporting bias with an asymetrical funnel plot suggesting smaller studies with high sensitivity are over represented (figure 7).

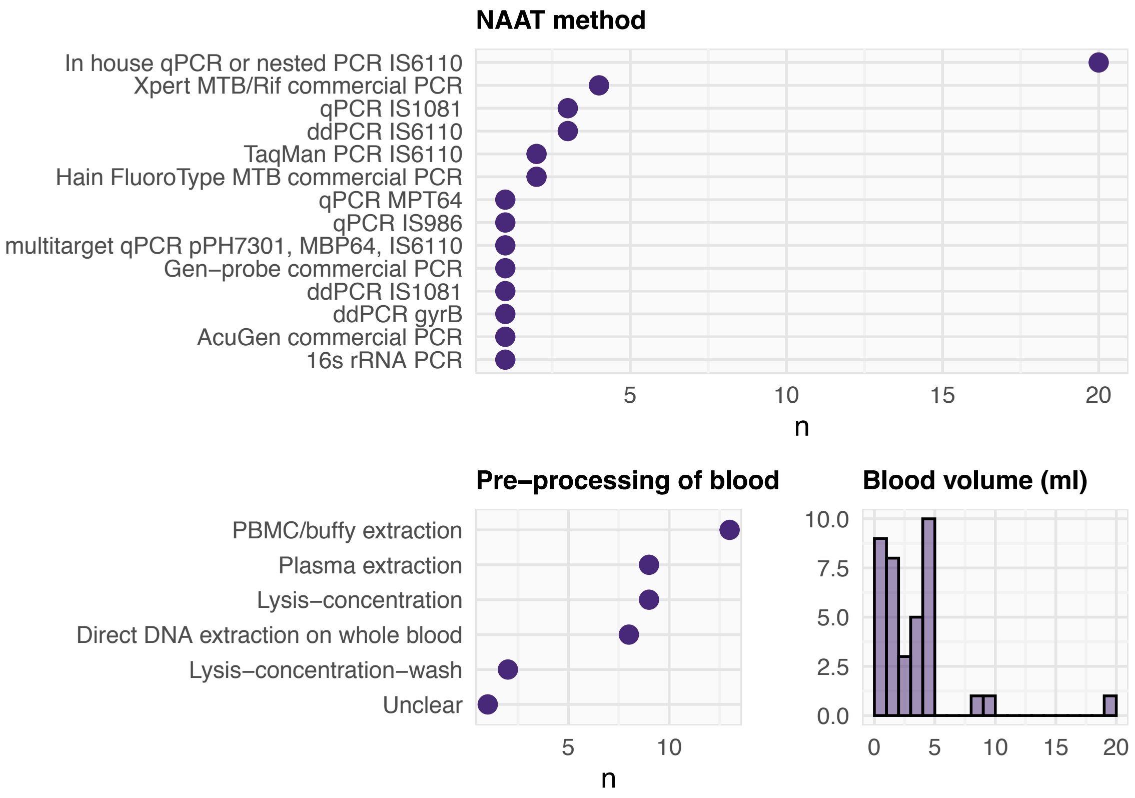

Figure 3: Description of review 1 included studies: NAAT methods used.

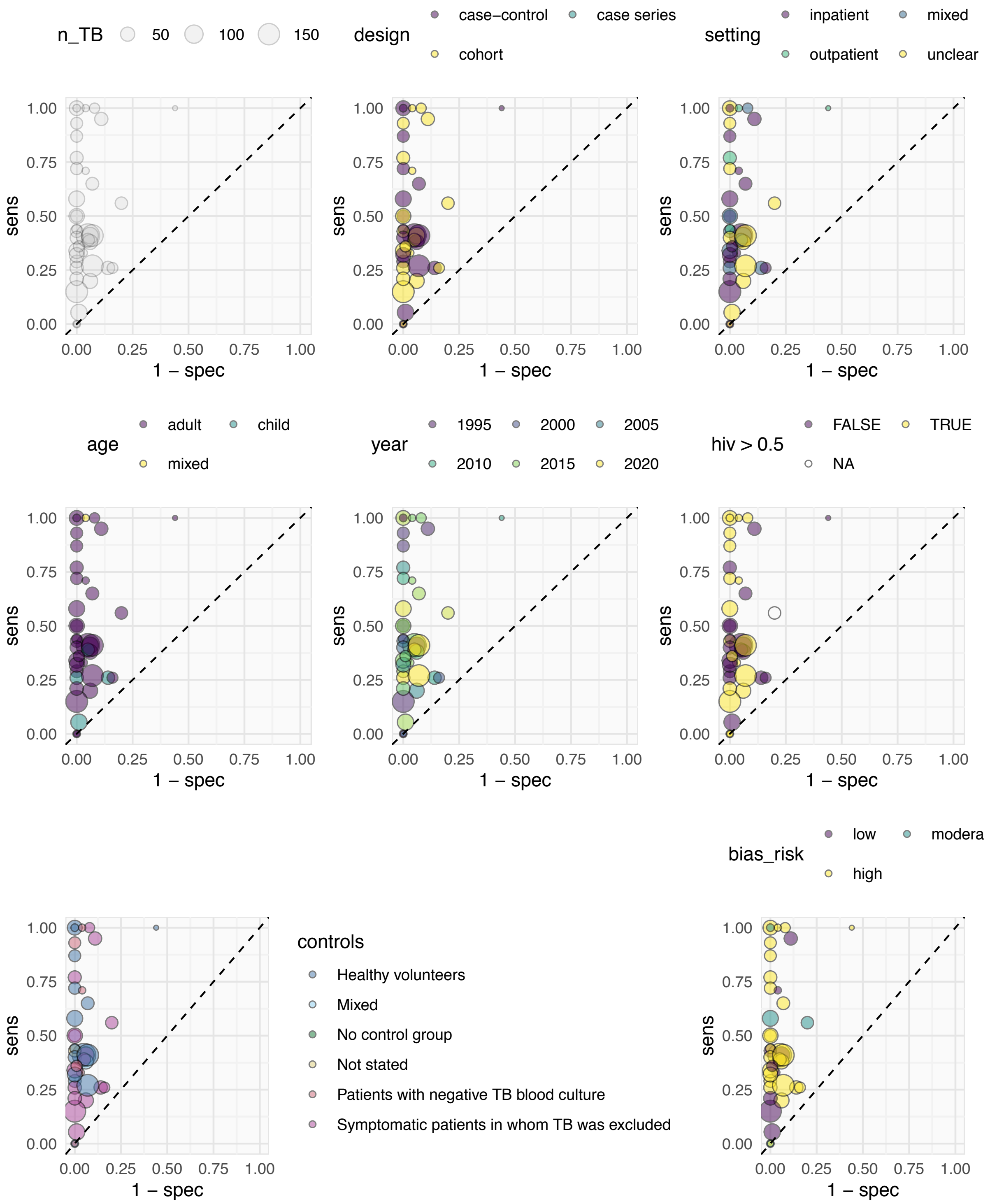

Figure 4: Reported diagnostic performance (sens v 1-spec, blood NAAT for TB diagnosis, raw data, review 1) by study characteristic covariates

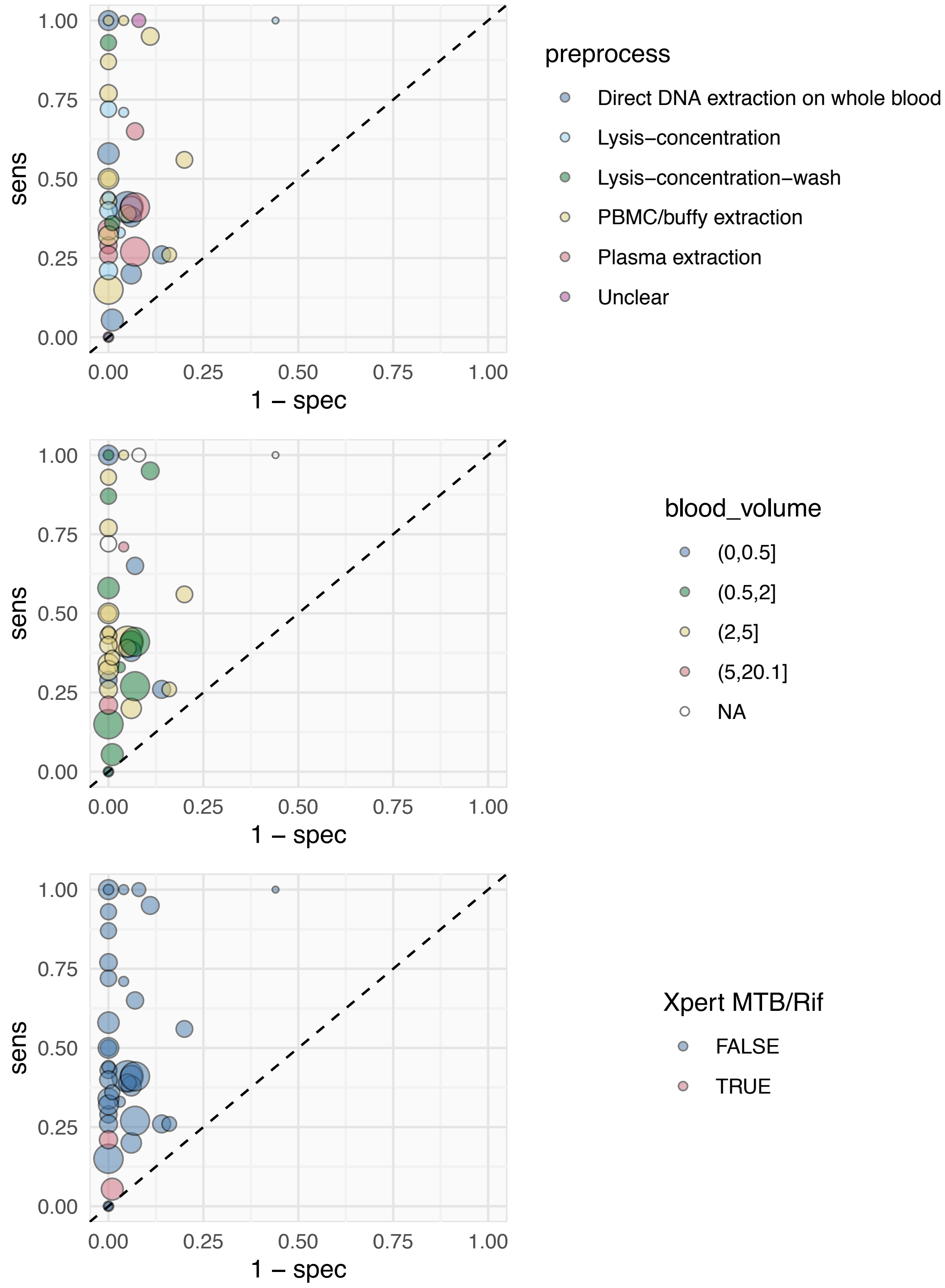

Figure 5: Reported diagnostic performance (sens v 1-spec, blood NAAT for TB diagnosis, raw data, review 1) by study NAAT methodology

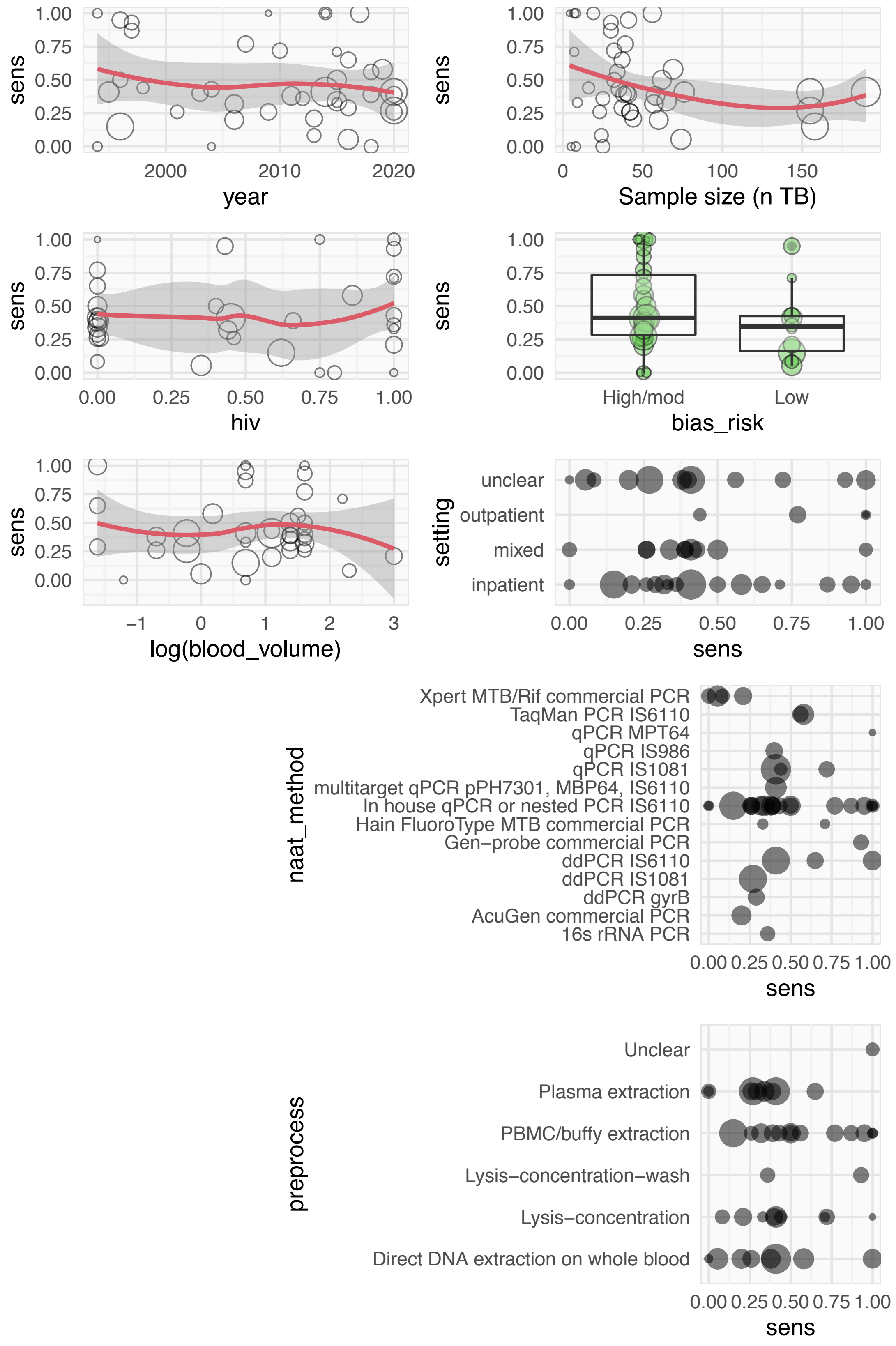

Figure 6: Reported sensitivity of blood NAAT for TB diagnosis (review 1, raw data) by selected covariates

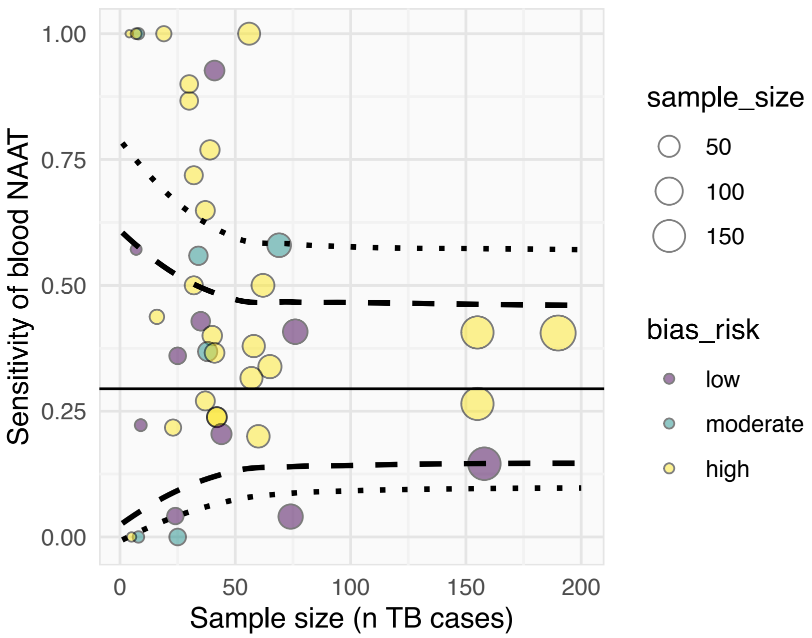

Figure 7: Evidence of bias in design and reporting versus reported sensitivity of blood NAAT for TB diagnosis (review 1).

2.6 Bivariate regression modelling

Formal bivariate modelling of the reported sensitivities and specificities largely confirms impressions from descriptive plots in previous section. Heterogeneity in reported sensitivity is extreme, with the 90% prediction interval (in which the model estimates 90% of studies from the "population" of studies will lie) encompassing nearly all possible values of sensitivity (90%PI = 0.074 to 0.97).

2.6.1 Meta-regression: covariates versus diagnostic performance

Association of study-level covariates with reported diagnostic performance of blood NAAT was formally investigated using bivariate random-effects modelling.

These was some evidence that reported sensitivity was associated with risk of bias assessed through adapted QUADAS-2: studies assessed as low risk of bias reported lower sensitivity on average (figure 9). Posterior probability that low-bias risk studies had lower reported sensitivity than 'high or moderate' bias risk studies was 94%.

There was also a 95% posterior probability that larger sample size studies reported lower sensitivity than smaller sample size studies (figure 10).

There was no significant evidence that reported sensitivity for TB diagnosis was improving over time as NAAT technologies have evolved (rather there was a 'non-significant' weak downward trend, with 73% posterior probability that sensitivity was *decreasing* by year of publication (figure 10).

Proportion of study participants who were HIV positive was not convincingly related to reported diagnostic performance of NAAT with 68% posterior probability that sensitivity was higher in studies recruiting more patients who were HIV positive (figure 10).

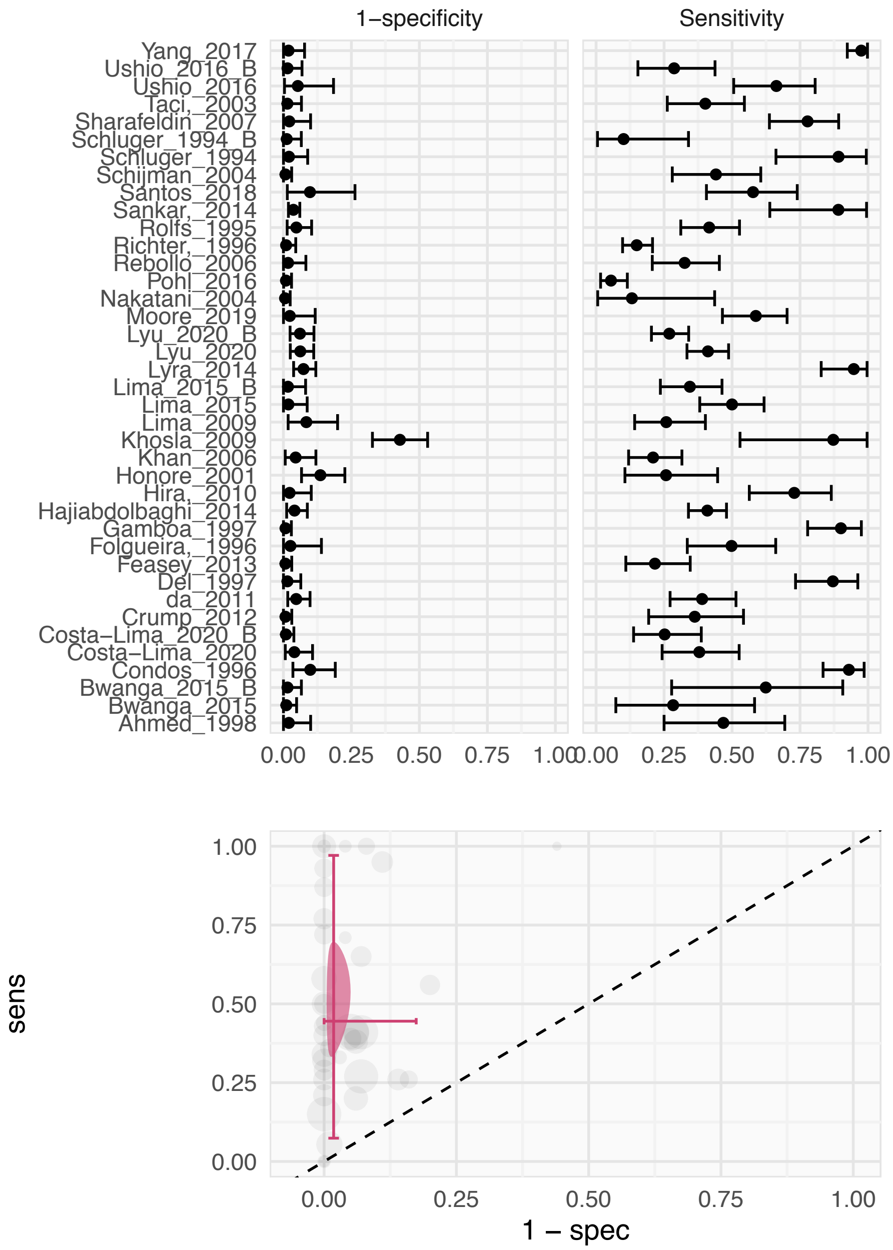

Figure 8: Bivariate random-effects modelling reported sensitivity and specificity blood NAAT for TB diagnosis (review 1). Forest plots show model estimates of mean values with 95% credibility intervals for each study. Lower plot shows 95% credibility ellipse for mean (expected) values, with 90% prediction intervals in which 90% of studies are expected to lie.

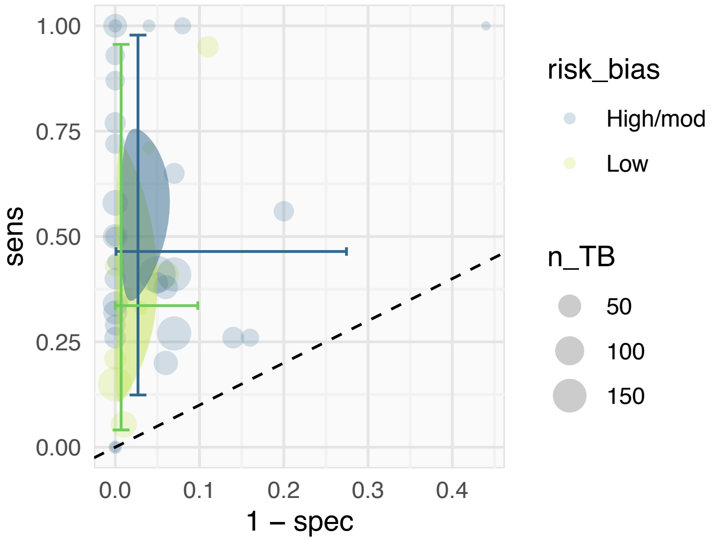

Figure 9: Bivariate random-effects model with assessed risk of study bias as covariate (high/moderate bias = blue; low bias = green) for performance of blood NAAT for TB diagnosis (review 1). Ellipses show 95%CrI for mean (expected) values; 90% prediction intervals shown with whisker intervals.

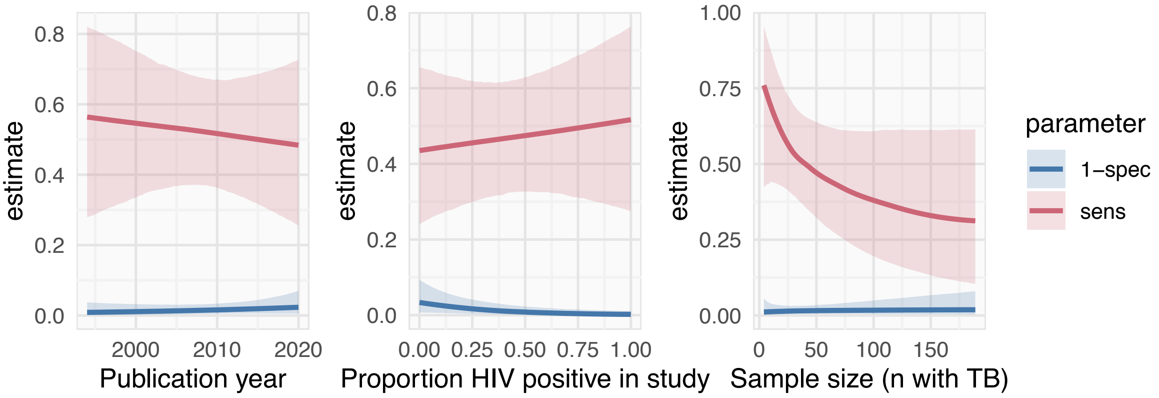

Figure 10: Conditional effects of 3 study-level covariates on reported performance in blood NAAT studies for TB diagnosis (review 1).

2.7 Summary review 1

Since the 1990s dozens of reports describing use of NAAT on patient blood samples for TB diagnosis have been published, with extreme heterogeneity in reported sensitivity and specificity, not obviously related to plausible biological or technical covariates. Most studies are poorly reported and are assessed to have high risk of bias. Mostly, in house PCR methods have been used with a wide variety of specific methodologies. Promising results in smaller reports have not been replicated in larger, low-bias studies. Results from scalable / widely available commercial PCR platforms have been disappointing.

3 Systematic Review 2: *M. tuberculosis* blood stream infection diagnosis using blood NAAT

3.1 Introduction

Marked heterogeneity in sensitivity of blood NAAT to diagnose tuberculosis could be related to disease spectrum of included TB cases in studies, not captured by gross study-level covariates such as inpatient v outpatient and proportion of patients HIV positive examined in review 1 above. We know that severity of HIV-associated tuberculosis is closely related to presence of *M. tuberculosis* blood stream infection (MTBBSI) and the presence of MTBBSI is clearly a plausible determinant of probability that TB is detected by blood NAAT testing. Detection of MTBBSI by blood culture is therefore a useful reference standard against which blood NAAT detection of tuberculosis can be assessed. We hypothesised that comparing blood NAAT to blood culture for detection of *M.tb* could resolve some of the heterogeneity in reported sensitivity by accounting for variance in disease spectrum of recruited cases in different studies, and therefore proposed a second systematic review limited to studies which peformed both blood NAAT and blood culture for detection of MTBBSI.

3.1.1 Aims and objectives

Objective is to summarise reported sensitivity of blood NAAT compared to mycobacterial blood culture.

Aims:

1. Summarise reported relative sensitivity of blood TB-NAAT and TB blood culture against an external reference standard.
2. Summarise reported sensitivity of blood TB-NAAT against TB blood culture as the reference standard. Note that specificity estimation is not an objective: this is justified as heterogeneity in sensitivity in systematic review 1 is the problem to be addressed.

3.1.2 Included studies

Included studies will be the subset of studies in review 1 that also performed a TB blood culture (liquid or solid media).

Studies which do not report results such that a 2x2 table cross-tabulating blood culture and NAAT results could be extracted were excluded.

[post hoc protocol edit:] *Studies where the TB reference standard was based on TB blood culture were excluded for aim 1 but were retained in aim 2 analysis].*

3.1.3 Data for extraction

1. **n__tb__diagnosis** : Number of patients who were classified as having TB diagnosis who had a valid TB blood culture and a blood NAAT performed.
2. **n__bloodculture** : Number of TB patients who were TB blood culture positive.
3. **n__bloodnaat** : Number of TB patients who were TB blood NAAT positive.
4. **n__bc__naat** : Number of TB blood culture positive patients who were also blood NAAT positive.

In addition to covariates assessed in review 1.

3.1.4 Analysis plan

Descriptive summaries using figures and tables.

For aim 1 (*Summarise reported relative sensitivity of blood TB-NAAT and TB blood culture against an external reference standard*) we anticipate correlation between reported sensitivity of blood culture and blood NAAT by study. Therefore a bivariate random-effects regression acounting for this correlation will be used to summarise central tendancies and heterogeneity. Ratio or difference measures for sensitivity of the two methods will then be derived.

For aim 2 (*Summarise reported sensitivity of blood TB-NAAT against TB blood culture as the reference standard*) a univariate binomial regression model with random-effects by study will be used to summarise central tendancies and heterogeneity.

Models will be fit using a Baysian (MCMC) approach implemented with the package *brms* in R studio. Meta-regression on selected covariates will be performed using bivariate regression to test association between study and method covariates and diagnostic performance if sufficient studies are identified to do so.

3.2 Screening results

From the 42 studies identified for review 1, 16 performed a mycobacterial blood culture; 2 of these did not report the results such that a 2x2 cross-tabulation of blood culture and blood NAAT could be extracted, leaving 14 studies with both blood culture and blood NAAT for inclusion in review 2 (figure 11, PRISMA flow chart). Of these 14 studies, 5 used TB blood culture result as the reference standard for TB diagnosis, meaning n=9 studies were available for aim 1. In one study all TB blood cultures were negative, meaning n=13 studies were available for aim 2 analysis.

3.3 Comparing sensitivity of blood TB-NAAT and TB blood culture against an external reference standard

In studies reporting sensitivity of blood NAAT and bood culture for TB diagnosis against an external reference standard there was, as expected, correlation between the sensitivities of the two methods across studies (r = 0.48 estimated from bivariate mixed-effects regression). Most studies reported higher sensitivity of NAAT compared to culture, but with substantial heterogeneity resulting in uncertainty and 95% credibility intervals encompassing both better and worse sensitiviy for NAAT (figure 12). Larger sample size and lower risk-of-bias studies reported lower relative sensitivity of NAAT on average, but the number of studies available did not support formal meta-regression.

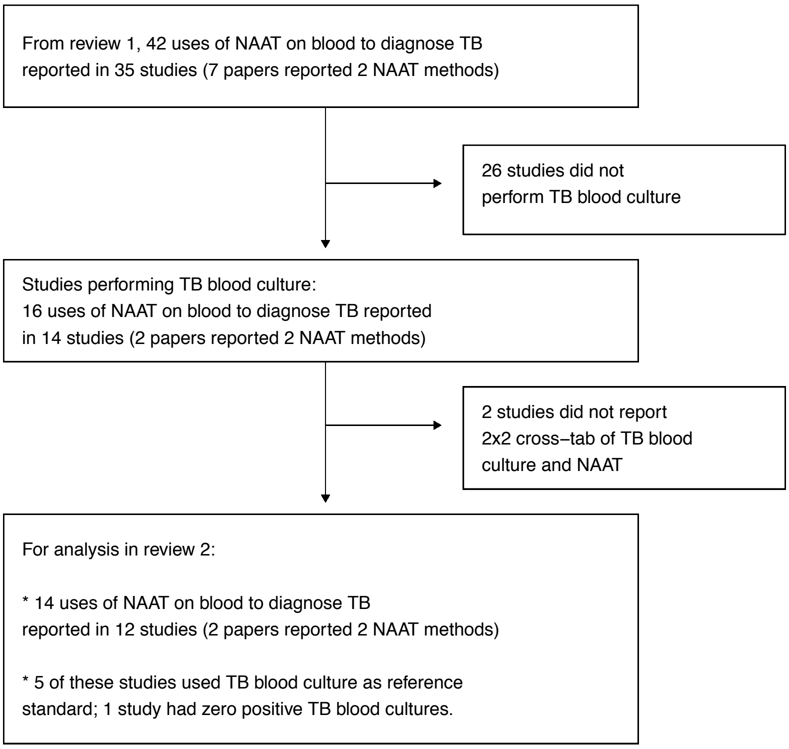

Figure 11: PRISMA flow chart for systematic review 2

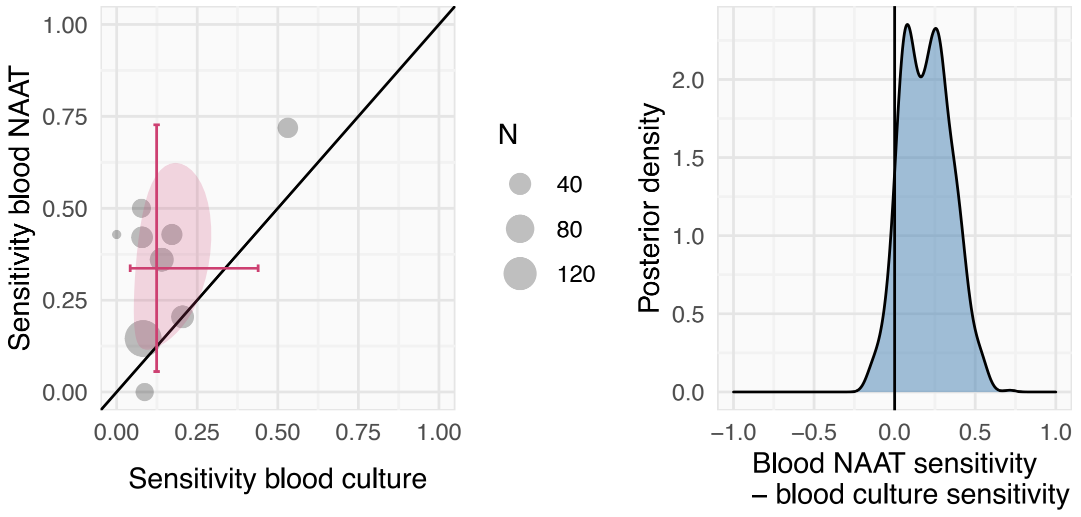

Figure 12: Bivariate mixed-effects regression relative sensitivity of TB blood culture and blood NAAT versus an external reference standard for TB diagnosis (review 2, aim 1). Left panel shows individual studies raw data for sensitivities (grey circles) and the 95%CrI for the median population value (red shaded area) and 90% prediction intervals for a new unobserved study (red lines). Right panel shows distribution of posterior estimates from the model for difference in sensitivity (estimate for sensitivity of NAAT minus sensitivity of culture); most probability was asigned by the model to higher sensitivity of NAAT, to the right of vertical black line.

Table 1: Mixed-effects meta-regression blood NAAT sensitivity for detection of blood culture positive TB: estimated difference in sensitivity of blood NAAT for two levels of study level variables.

effect	comparator	delta sensitivity	95% CrI
low bias	high/moderate bias	-0.02	-0.6 to 0.66
year 2015	year 1995	-0.22	-0.7 to 0.55
commercial_kit	in house kit	-0.41	-0.92 to 0.28
Study size, N=50	Study size, N=5	0.00	-0.02 to 0.02

3.4 Assessing sensitivity of blood TB-NAAT against TB blood culture as the reference standard

13 studies have reported TB blood culture and TB blood NAAT results in same patients. Estimated sensitivity of TB blood NAAT for TB blood culture cases (population median across all 13 studies, figure 13) was 0.7, but with substantial uncertainty for this population estimate (95% CrI 0.39 to 0.94, and 90% prediction interval for a new, unobserved study 0.07 to 0.99) due to heterogeneity and the limited amount of published data (in total, blood TB NAAT results have only been reported for 174 TB blood culture positive patients, with median of 7 patients per study).

This means there is limited power to support meta-regression. More recent studies, studies using commercial NAAT kits, and studies assessed to be at lower risk of bais all had lower reported sesnitivity of NAAT for TB blood culture positive disease but none of these associations reached a >95% posterior probability statistical significance level (table 1).

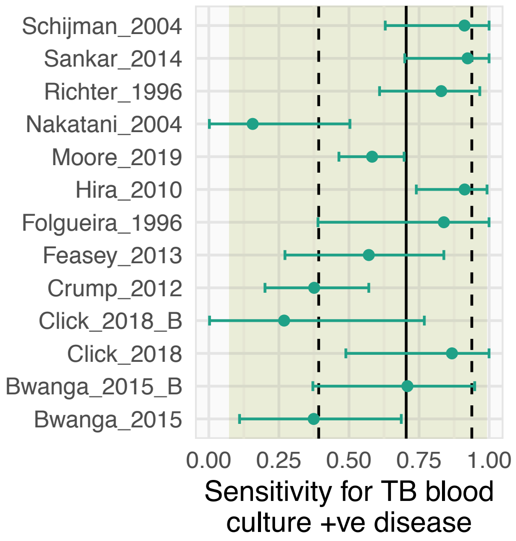

Figure 13: Mixed-effects regression blood NAAT sensitivity for detection of blood culture positive TB (review 2, aim 2). Fit and 95%CrI for individiual studies shown with green dots and whisters; estimated population median and 95%CrI shown with vertical solid and dashed lines respectively; 90% prediction intervals for a new unobserved study indicated by shaded green area.

3.5 Summary review 2

Published data on blood NAAT for TB diagnosis where sensitivity can be related to a concomitant TB blood culture is sparse. This data was reviewed because sensitivity of blood TB NAAT relative to TB blood culture allows a degree of adjustment for disease spectrum which we hypothesised might underlie the extreme heterogeneity described in review 1. However, variance in reported sensitivity of TB blood NAAT was still pronounced relative to TB blood culture and within the strata of patients who were TB blood culture positive. Reported sensitivity of TB blood NAAT was again lower on average in low bias rated studies, studies using commercial NAAT kits, and in more recently reported studies compared to initial reports in 1990s; these associations were not statistically significant, which may relate to the limited amount of data available in review 2.
